# Inhibition of Hepatitis B Virus Replication by the Host Zinc Finger Antiviral Protein

**DOI:** 10.1371/journal.ppat.1003494

**Published:** 2013-07-11

**Authors:** Richeng Mao, Hui Nie, Dawei Cai, Jiming Zhang, Hongyan Liu, Ran Yan, Andrea Cuconati, Timothy M. Block, Ju-Tao Guo, Haitao Guo

**Affiliations:** 1 Institute for Biotechnology and Virology Research, Department of Microbiology and Immunology, Drexel University College of Medicine, Doylestown, Pennsylvania, United States of America; 2 Key Laboratory of Medical Molecular Virology of the Ministries of Education and Health, Department of Infectious Diseases, Huashan Hospital, Fudan University, Shanghai, China; 3 Institute for Hepatitis and Virus Research, Hepatitis B Foundation, Doylestown, Pennsylvania, United States of America; University of California, San Diego, United States of America

## Abstract

The zinc finger antiviral protein (ZAP) is a mammalian host restriction factor that inhibits the replication of a variety of RNA viruses, including retroviruses, alphaviruses and filoviruses, through interaction with the ZAP-responsive elements (ZRE) in viral RNA, and recruiting the exosome to degrade RNA substrate. Hepatitis B virus (HBV) is a pararetrovirus that replicates its genomic DNA via reverse transcription of a viral pregenomic (pg) RNA precursor. Here, we demonstrate that the two isoforms of human ZAP (hZAP-L and -S) inhibit HBV replication in human hepatocyte-derived cells through posttranscriptional down-regulation of viral pgRNA. Mechanistically, the zinc finger motif-containing N-terminus of hZAP is responsible for the reduction of HBV RNA, and the integrity of the four zinc finger motifs is essential for ZAP to bind to HBV RNA and fulfill its antiviral function. The ZRE sequences conferring the susceptibility of viral RNA to ZAP-mediated RNA decay were mapped to the terminal redundant region (nt 1820–1918) of HBV pgRNA. In agreement with its role as a host restriction factor and as an innate immune mediator for HBV infection, ZAP was upregulated in cultured primary human hepatocytes and hepatocyte-derived cells upon IFN-α treatment or IPS-1 activation, and in the livers of hepatitis B patients during immune active phase. Knock down of ZAP expression increased the level of HBV RNA and partially attenuated the antiviral effect elicited by IPS-1 in cell cultures. In summary, we demonstrated that ZAP is an intrinsic host antiviral factor with activity against HBV through down-regulation of viral RNA, and that ZAP plays a role in the innate control of HBV replication. Our findings thus shed light on virus-host interaction, viral pathogenesis, and antiviral approaches.

## Author Summary

The dynamics of virus and host interaction greatly influence viral pathogenesis, and host cells have evolved multiple mechanisms to inhibit viral replication. Since it was first discovered as a cellular restriction factor for retroviruses, the host-encoded zinc finger antiviral protein (ZAP) has been shown to antagonize a variety of viral species, possibly through a common mechanism by which ZAP targets viral RNA for degradation. Here we report that hepatitis B virus (HBV) is also vulnerable to ZAP-mediated viral RNA reduction. ZAP is able to interact with HBV RNA through its zinc finger motifs, and the ZAP-responsive element which determines ZAP's antiviral specificity and activity is located within the 100-nucleotide-long terminal redundant region in the viral RNA genome. While the replication of HBV is constitutively restricted under the basal expression of intrahepatic ZAP, activation of host innate defenses, and potentially the acquired immune responses as well, could further elevate ZAP expression to suppress HBV replication. Therefore, our study not only expands the antiviral spectrum of ZAP, but also provides cumulative and novel information for a better understanding of ZAP biology and antiviral mechanisms. We also envision that the endogenous or engineered ZAP could be utilized in the future for development of therapeutic means to treat chronic hepatitis B, which currently affects more than 5% of the world's population.

## Introduction

Hepatitis B virus (HBV) is the etiological agent of human hepatitis B. Despite the fact that most adulthood HBV infections are transient, approximately 5–10% of infected adults and more than 90% of infected neonates develop a life-long chronic infection, constituting a substantial public health burden affecting an estimated 350 million individuals worldwide. HBV carriers suffer from a high risk of cirrhosis, primary hepatocellular carcinoma, and other severe clinical sequelae [1]–[5].

HBV is a noncytopathic, hepatotropic virus belonging to the *hepadnaviridae* family. The virion is an enveloped icosahedral nucleocapsid containing a partially double stranded relaxed circular (RC) DNA genome of 3.2 kb. Upon infection of hepatocytes, the viral RC DNA enters the nucleus and converts into an episomal covalently closed circular (ccc) DNA, which serves as the template for all viral RNA transcripts, including precore mRNA (3.5∼3.6 kb), pregenomic (pg) RNA (3.5 kb), surface (envelope) mRNA (2.4 and 2.1 kb), and X mRNA (0.7 kb). After nuclear export, cytoplasmic pgRNA is translated into viral capsid proteins and polymerase (pol), followed by *in situ* binding of pol to a stem loop structure termed epsilon (ε) at the 5′ terminus of pgRNA, which in turn triggers encapsidation of the pol/pgRNA complex. Viral double stranded DNA synthesis then occurs, inside of the nucleocapsid, in an asymmetric fashion. Viral pol reverse transcribes pgRNA into minus strand DNA, followed by plus strand DNA synthesis and circularization into the RC DNA genome. The mature cytoplasmic nucleocapsid is then packaged by viral envelope proteins and secreted as a progeny virus. Alternatively, the newly synthesized RC DNA can be transported to the nucleus to amplify the cccDNA reservoir, thereby maintaining a chronic state of HBV infection. (Reviewed in [2], [6], [7])

It is generally accepted that host functions determine virus tropism and replication fitness [8], [9]. HBV reproduction requires liver enriched transcription factors (i.e. HNF4 and RXRα) as well as more ubiquitous host factors, such as chaperon protein Hsp90 that coordinates the assembly of the pol/pgRNA complex [10]–[13]. The reproduction of HBV is also restricted by intrinsic and extrinsic host factors and stimuli. Specific hormones and inflammatory cytokines/chemokines have been shown to suppress HBV replication both in cultured cells and *in vivo* [14]–[20]. It is also clear that the balance between viral replication and host immunity determines the course of viral infection. Rigorous HBV specific polyclonal cytotoxic T lymphocytes and humoral responses can clear viral infection, whereas the lesser responses can cause chronic liver disease without actually eliminating the virus [21], [22]. In coordination with adaptive immune responses, host innate immunity also plays a critical role in the control of viral replication [23]. Central to this cellular response is the secretion of interferons (IFN-α/β), which act on target cells to induce an array of interferon stimulated genes (ISGs) that limit virus infection [24]. Although considerable evidence suggests that HBV has evolved strategies to evade or antagonize host innate defenses [25]–[29], viral replication is still somewhat suppressed by IFN in laboratory studies and clinical treatments [15], [16], [30], [31]. The intracellular antiviral response elicited by IFN targets multiple steps in the HBV life cycle, including HBV RNA synthesis [31]–[33], pgRNA encapsidation [34], and the turnover rate of viral proteins and nucleocapsids [35], [36]. Therefore, it is of interest to identify host proteins that impede HBV replication under homeostatic or inducible conditions.

The zinc finger antiviral protein (ZAP, also known as ZC3HAV1) was originally discovered in rat as a host antiretroviral factor that prevents cells from infection by Moloney murine leukemia virus (MMLV) [37]. The antiviral spectrum of rat ZAP (rZAP) has been expanded to other kinds of RNA viruses, including certain alphaviruses (Sindbis virus and Ross River virus) and filoviruses (Ebola virus and Marburg virus) [38]–[40]. Human ZAP exists in long and short isoforms (hZAP-L and hZAP-S, respectively) derived from alternative mRNA splicing, with hZAP-L containing an additional C-terminal poly(ADP-ribose) polymerase (PARP) domain [41]. Both hZAP isoforms have antiviral effects against several RNA viruses, including MMLV, Semliki Forest virus, HIV-1 and xenotropic murine leukemia virus-related virus (XMRV) [41]–[43]. However, rZAP does not inhibit the replication of vesicular stomatitis virus, yellow fever virus, poliovirus, or herpes simplex virus 1, indicating that the antiviral activity of ZAP is highly reliant on virus-specific features [38]. Along these lines, mechanistic studies revealed that rZAP binds to its viral RNA substrate through its N-terminal zinc finger motifs, and in turn recruits a host RNA processing complex, specifically the exosome, to degrade the viral RNA [44]–[46]. The viral RNA motif responsible for the binding of ZAP is called the ZAP-responsive element (ZRE), which has been mapped to the 3′-LTR of MMLV [44], 5′-UTR of HIV-1 nef mRNA [42], 3′-UTR of XMRV [43], and multiple fragments in Sindbis virus [44]. Although these ZREs are all more than 500 nucleotides long, there is no significant degree of sequence homology or common structural similarity in these ZREs [42]–[44], [46]. Recently, ZAP was shown to inhibit murine gammaherpesvirus 68 (MHV-68) M2 mRNA expression [47]. This is the first report that ZAP possesses antiviral activity against a DNA virus. Since mRNA intermediate(s) is an essential component for DNA viruses to fulfill their life cycle, it is possible that ZAP might specifically target certain DNA virus encoded mRNA for degradation to inhibit virus replication.

Based upon a defining feature, that a reverse transcription step is involved in hepadnavirus DNA replication, HBV has been classified as a member of the pararetroviruses, a groups of viruses which have evolutionary relationships with retroviruses [7], [48]. As a well-known antiretroviral host factor, it is of interest to examine whether ZAP also restricts HBV replication. In addition, cellular ZAP mRNA can be upregulated under interferon treatment or certain virus infection, suggesting ZAP may play a role in the innate defenses of virus infection [49]–[52]. Moreover, previous reports suggested that HBV mRNA down-regulation is involved in the antiviral state elicited by the activation of innate immune response [32], [53]. Therefore, we also studied the potential function of ZAP in the innate control of HBV replication.

Herein, we report that ectopic expression of ZAP can markedly suppress HBV replication in human hepatocyte-derived cells, primarily through posttranscriptional down-regulation of viral RNA in the cell nuclei. The zinc-finger-motifs-containing N-terminus is the functional antiviral domain of ZAP, and the integrity of the tetra-partite zinc fingers confers the optimal antiviral activity against HBV. Furthermore, we observed a physical interaction between ZAP and HBV RNA in the immunoprecipitation assay, and the HBV ZRE was mapped to the terminal redundant region of viral RNA. In addition, both hZAP-L and hZAP-S exhibit a basal level expression in primary human hepatocytes and hepatocyte-derived cells, and the levels of the short isoform could be further induced by IFN treatment or expression of beta interferon promoter stimulator 1 (IPS-1). Knock down experiment revealed that the basal level of ZAP limits HBV replication, and ZAP plays a partial role in IPS-1-induced HBV RNA destabilization. Our results thus demonstrated that ZAP is a host intrinsic antiviral factor against HBV, and suggested that ZAP plays a role in the innate control of HBV replication. The inclusion of HBV in ZAP's antiviral spectrum will not only contribute to a better understanding of ZAP biology and virus-host interaction, but may ultimately lead to development of novel strategies utilizing host functions for hepatitis B therapeutics.

## Results

### Overexpression of ZAP inhibits HBV replication in cell cultures

**Figure 1:**
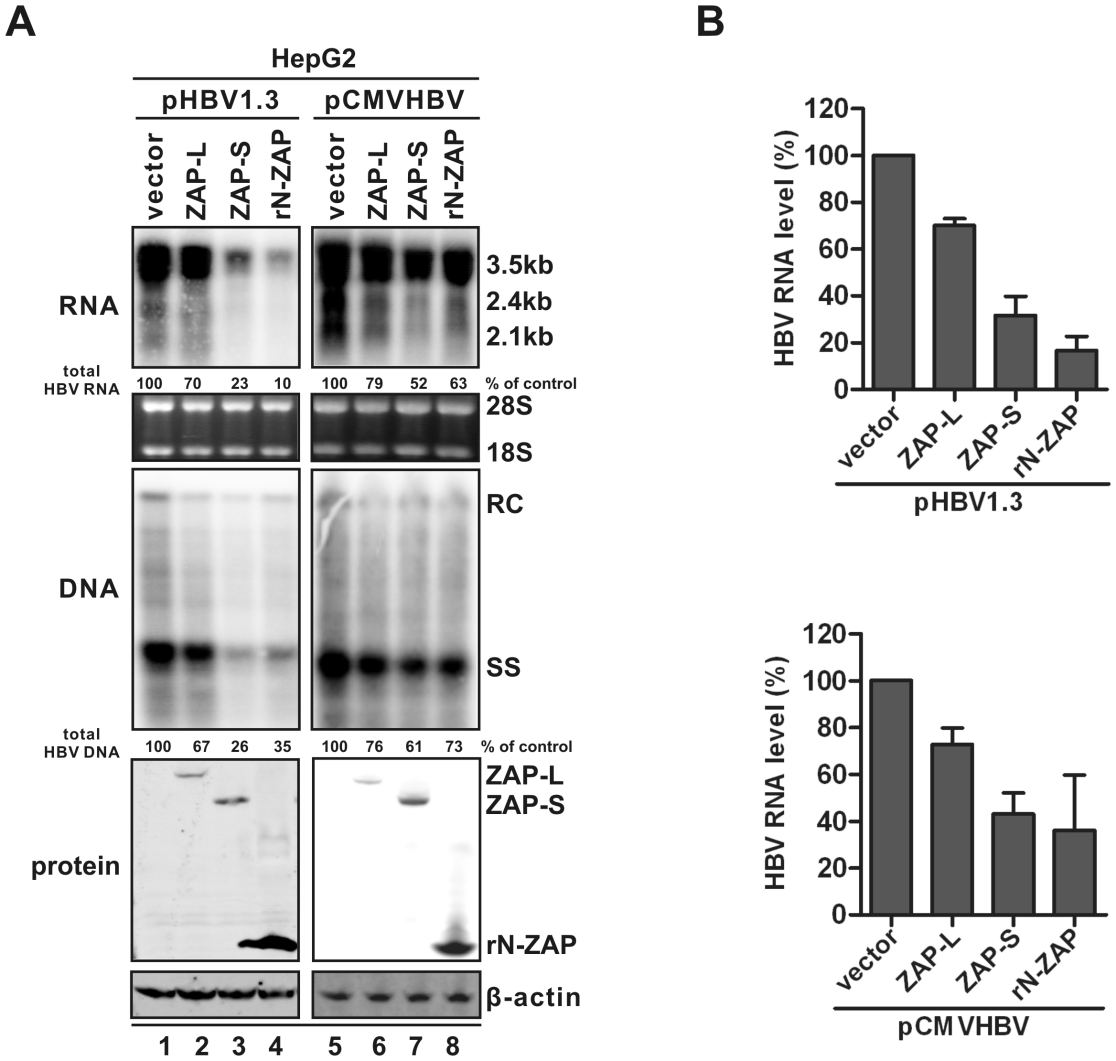
Inhibitory effects of ZAP on HBV replication in cell cultures. (A) HepG2 cells in 35 mm dishes were cotransfected with 2 µg of pHBV1.3 containing 1.3 mer genome-length HBV sequences (left panels), or 2 µg of pCMVHBV (right panels), in which viral pgRNA transcription is under the control of CMV-IE promoter, and 2 µg of control empty vector (lanes 1 and 5), or plasmids expressing HA-tagged hZAP-L (lanes 2 and 6), hZAP-S (lanes 3 and 7), and rN-ZAP (lanes 4 and 8), respectively. Cells were harvested at day 5 post transfection and viral RNA and core DNA were analyzed by Northern (upper panels) and Southern (middle panels) blot assays, respectively. For RNA analysis, each lane was loaded with 10 µg of total RNA and probed with a genome-length, plus strand specific HBV riboprobe. Ribosomal RNAs (28S and 18S) are presented as loading controls. The positions of HBV 3.5 kb, 2.4 kb, and 2.1 kb RNAs are indicated. For DNA analysis, HBV core DNA was probed with a genome-length, minus strand specific HBV riboprobe. The positions of relaxed circular (RC), single-stranded (SS) DNAs are indicated. Relative viral RNA or DNA level in each sample was expressed as the percentage of RNA or DNA in control cells (lane 1 and 5), and was indicated underneath each of the blots. Expression of ZAP was revealed by Western blot analysis with HA antibodies. The levels of β-actin served as a loading control (Lower panels). (B) Experiments shown in (A) were repeated in triplicate. Viral RNA levels were quantified and plotted as relative level (mean ± SD) of control samples.

As shown in Fig. 1A, overexpression of both human ZAP (-L or -S) and the N-terminus of rat ZAP (rN-ZAP) led to a significant reduction of HBV DNA replication in HepG2 cells (middle panels). This was achieved primarily through reducing the steady state levels of viral pgRNA (top panels), which is the template for HBV DNA synthesis. The doublet bands of pgRNA contain the full-length HBV pgRNA of 3.5 kb (lower band) and a longer form of pgRNA transcripts (upper band) terminated at an additional polyadenylation site in the vector sequences from pHBV1.3 or pCMVHBV (data not shown). The less significant inhibitory effect against HBV replication by ZAP-L was likely due to its lower level of expression (bottom panel). Both hZAP-L and -S exhibited antiviral activity, indicating that the C-terminal PARP domain in hZAP-L is dispensable. Reduction of HBV RNA by ZAP expression was independent of the promoter driving transcription of HBV pgRNA (HBV authentic core promoter in pHBV1.3, CMV-IE promoter in pCMVHBV), although an apparent lower degree of inhibition in the CMV-IE driven samples was likely due to overall higher RNA expression levels and ZAP may work stoichiometrically, which the ratio between the amount of HBV RNA and ZAP determines the degree of inhibitory effect of ZAP. The statistical analyses of ZAP-mediated HBV RNA reduction from multiple repeated experiments are depicted in Fig. 1B. These observations indicate that ZAP may posttranscriptionally promote HBV pgRNA decay, or perhaps inhibit a common transcription factor(s) shared by both viral promoters.

ZAP's antiviral activity against HBV was not limited to HepG2 or hepatocyte-derived cells. Expression of ZAP also efficiently reduced HBV RNA in hepatoma Huh7 cells and in human embryonic kidney 293T cells (Fig. S1), suggesting that liver-specific host factors are not absolutely required for ZAP's antiviral function.

In addition, along with pgRNA reduction, the levels of 2.4 kb and 2.1 kb HBV mRNA, which share 100% sequence identity with the 3′ portion of pgRNA, were also reduced upon ZAP expression (Fig. 1 and S1). Considering that the two subgenomic RNA entities might contain both surface antigen mRNA and putative spliced pgRNA [54], we further demonstrated that ZAP-S was able to reduce viral surface mRNAs in Huh7 cells transfected with vectors expressing them alone (Fig. S2).

### ZAP down-regulates HBV RNA via posttranscriptional mechanisms

In order to determine whether ZAP-mediated down-regulation of HBV RNA was due to a transcriptional or posttranscriptional mechanism, we first ruled out the possibility that ZAP may degrade the transfected HBV plasmids. As shown in Fig. S3, while ZAP-S expression reduced the levels of HBV DNA replication, the cotransfected HBV plasmid signal revealed by Dpn I digestion and DNA hybridization was equivalent to the control (Fig. S3A). In addition, overexpression of ZAP-S reduced viral RNA and DNA in the HBV stable cell line HepDES19, in which HBV RNA is transcribed from an integrated transgene (Fig. S3B). Collectively, these observations suggested that ZAP expression does not alter the amount or stability of HBV RNA transcription templates.

**Figure 2:**
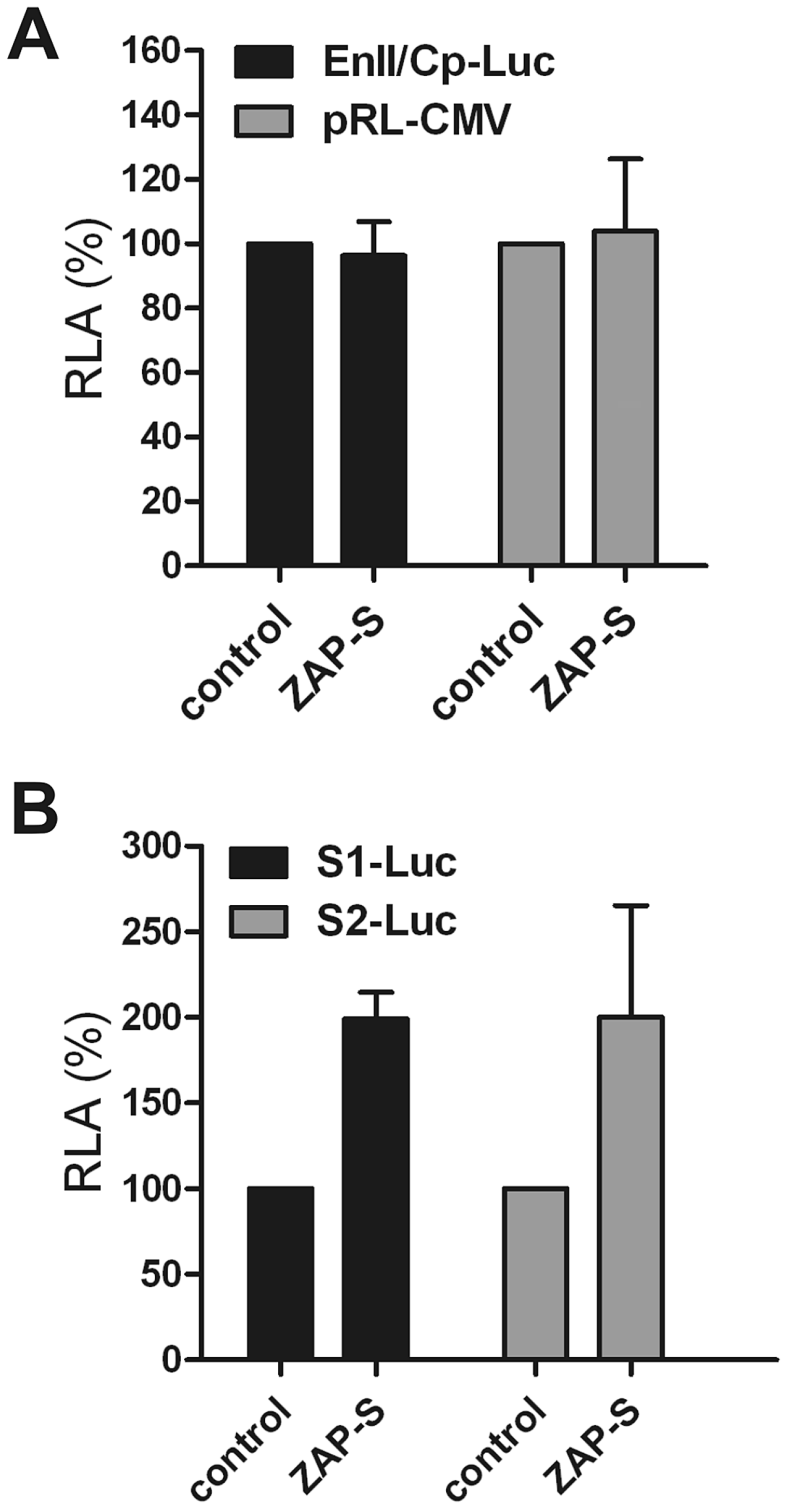
Effect of ZAP-S on viral promoter activities. (A) Expression of ZAP-S does not alter HBV core promoter activity in transfected cells. HepG2 cells were seeded in a 96-well-plate and cotransfected with 100 ng of EnII/Cp-Luc and 4 ng of pRL-CMV, plus 100 ng of control vector or plasmid ZAP-S. Three days after transfection, cells were harvested and luciferase activities were measured. The plotted relative luciferase activity (RLA) represents the mean ± standard deviation (SD, n = 4) of the ratios of absorbance obtained from wells expressing ZAP-S over that obtained from wells that were transfected with control vector. (B) ZAP-S overexpression enhances HBV surface promoter activity. HepG2 cells in 96-well-plate were transfected with 100 ng of S1-Luc or S2-Luc, together with 100 ng of control plasmid or ZAP-S expressing vector. 4 ng of pRL-CMV was included in each transfection for the normalization of transfection efficiency. Luciferase assays were performed 3 days post transfection.

Next, promoter reporter assays demonstrated that ZAP-S did not significantly affect the activities of HBV core promoter and CMV-IE promoter (Fig. 2A), and even boosted HBV S1 and S2 promoter activities (Fig. 2B). Thus, we speculated that ZAP-mediated HBV RNA reduction was not due to a transcriptional inhibition, if any, but rather through accelerating HBV RNA decay.

**Figure 3:**
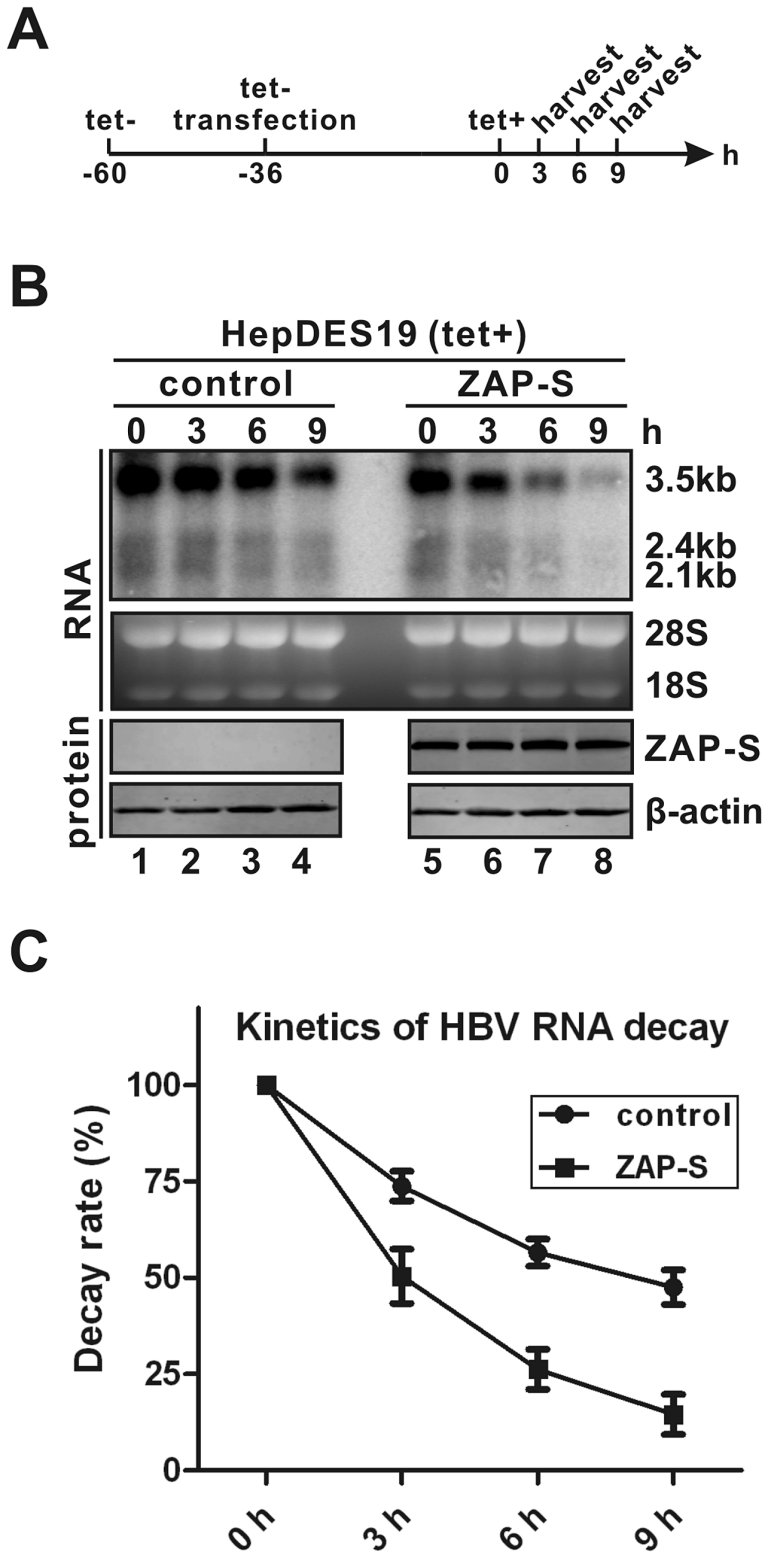
ZAP-S promotes HBV RNA decay in cell cultures. (A) Experimental procedure: HepDES19 cells were seeded in 35 mm-dish and cultured with tetracycline-free medium to induce HBV RNA expression. One day later, cells were transfected with 4 µg of control vector or plasmid ZAP-S for 36 h, then tetracycline was added back to the culture medium to shut down pgRNA transcription. Cells were harvested at indicated time points. (B) HBV RNA was extracted from harvested samples and analyzed by Northern blot. Expression of HA-tagged ZAP-S was detected by Western blot. The results are representative of three separate trials. (C) Kinetics analysis of HBV RNA decay in the absence or presence of ZAP-S overexpression. The relative levels of HBV RNA from each sample were expressed as the percentage of the RNA signals from the corresponding sample at time point 0 h.

To further confirm the above hypothesis, we directly measured the decay kinetics of HBV RNA in the absence and presence of ZAP overexpression. Briefly, HepDES19 cells were transfected with control vector or plasmid ZAP-S in the absence of tetracycline (to induce HBV RNA transcription); after 36 h, tetracycline was added back to the medium to shut down *de novo* transcription of HBV pgRNA from the transgene, and the decay kinetics of HBV RNA were determined in a time course study. As shown in Fig. 3B–C, the velocity of HBV RNA degradation in HepDES19 cells was faster in the presence of overexpressed ZAP-S than that in the control experiment, suggesting that ZAP promotes HBV RNA degradation. Thus, we concluded that ZAP-mediated HBV RNA reduction is through posttranscriptional downregulation of HBV RNA stability. This is consistent with the previous observations that ZAP reduced viral RNA in a posttranscriptional manner [37]–[39], [42].

### ZAP primarily promotes HBV RNA decay in the nucleus

**Figure 4:**
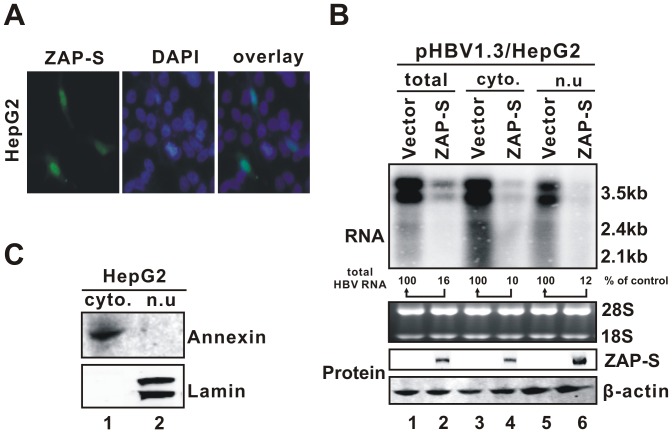
ZAP-mediated HBV RNA reduction primarily occurs in the nucleus. (A) Intracellular localization of ZAP-S by microscopic immunofluorescence analysis. HepG2 cells were transfected with HA-tagged ZAP-S expression plasmid. Cellular distribution of ZAP-S was stained with HA antibodies and corresponding fluorescence-labeled secondary antibodies (left panel). DAPI staining of nucleus is shown in the middle panel. Merged signals of ZAP-S and nucleus are shown in the right panel. (B) Subcellular distribution and antiviral activity of ZAP-S. HepG2 cells in 35 mm dishes were transfected with 2 µg of plasmid pHBV1.3 and 2 µg of ZAP-S expression vector. Cells were harvested at day 4 post transfection. Cell fractionations for RNA and protein analysis were performed as described in the Materials and Methods. Total cellular RNA, cytoplasmic and nuclear RNA were isolated and subjected to Northern blot analysis of HBV RNA (upper panel). Subcellular distribution of ZAP-S was revealed by Western blot using HA antibodies, with β-actin serving as loading control (lowers panels). Annexin and Lamin A/C Western blots were used to confirm the purity of cytoplasmic and nuclear fraction, respectively (panel C).

Previous studies showed that rat ZAP shuttles between the nucleus and the cytoplasm in a CRM1-dependent manner [55]. We first examined the intracellular distribution of hZAP-S in human hepatocyte-derived cells. Immunofluorescence microscopy (Fig. 4A) and cell fractionation experiments (Fig. 4B, C) demonstrated that hZAP-S was present in both cytoplasm and nucleus, and that hZAP-S-mediated HBV mRNA decay was primarily a nuclear event, although the possibility that ZAP also reduces HBV RNA in the cytoplasm cannot be ruled out. It has been previously shown that rN-ZAP prevented retroviral RNA accumulation in the cytoplasm without affecting the nuclear viral mRNA level in rat fibroblast cells [37]. The observed purge of HBV mRNA in the nucleus by hZAP-S indicated that the cell nucleus contains all the necessary machinery for ZAP-mediated RNA degradation, although this phenomenon may be specific to HBV and/or hepatocytes.

### The integrity of N-terminal zinc finger domain (ZFD) is required for ZAP's antiviral activity against HBV

**Figure 5:**
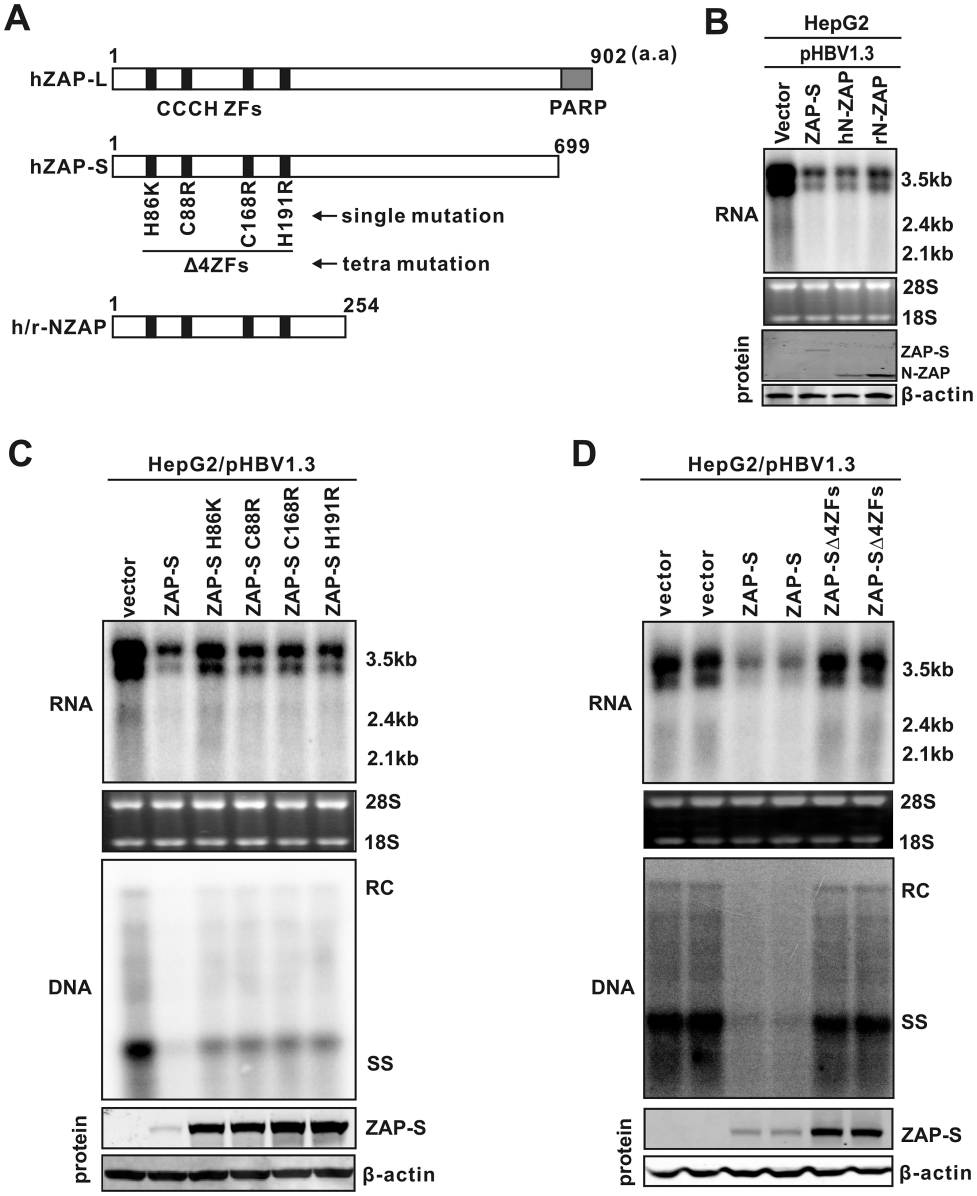
Mapping the functional antiviral domain and zinc finger motifs of ZAP. (A) Schematic structure of hZAP isoforms (hZAP-L, 902 a.a.; hZAP-S, 699 a.a.) and N-terminus of ZAP (human and rat, 254 a.a.). The gray box indicates the C-terminal PARP motif in hZAP-L. The positions of four tandem CCCH-type zinc finger (ZF) motifs within the N-terminus of ZAP are shown as solid black boxes. Mutations that disrupt each individual ZFs are indicated underneath hZAP-S and designated ZAP-S H86K, ZAP-S C88R, ZAP-S C168R, and ZAP-S H191R, respectively, according to previous studies [44]. ZAP-S that contains all the above four mutations is named as ZAP-SΔ4ZFs. (B) Expression of N-terminus of ZAP reduces the level of HBV RNA. HepG2 cells were cotransfected with pHBV1.3 and control plasmid, or full length ZAP-S, or N-terminal portion of human ZAP (hN-ZAP) or rat ZAP (rN-ZAP). Viral RNA and HA-tagged ZAP protein expression were analyzed at day 4 post transfection. (C) Disruption of each individual zinc fingers of ZAP partially reduces ZAP's antiviral activity. HepG2 cells were cotransfected with indicated plasmids. Viral RNA and DNA, and the expression of HA-tagged wildtype and mutant ZAP-S, were analyzed after 5 days post transfection. (D) Disruption of the full set of zinc fingers completely attenuates the antiviral activity of ZAP. HepG2 cells were transfected with pHBV1.3 and control plasmid, or wildtype ZAP-S, or ZAP-SΔ4ZFs. Five days later, viral nucleic acids and protein level of HA-tagged ZAP-S and ZAP-SΔ4ZFs were assayed by Northern and Southern hybridizations and Western blot, respectively. β-actin served as protein loading control. Results from duplicate experiments are presented.

ZAP contains four putative CCCH-type zinc finger motifs (ZF) in its N-terminal 254 amino acid (a.a.) portion, which is highly conserved among rat, mouse and human ZAP isoforms (Fig. 5A) [37], [41], [44], [56]. The conformation and spatial disposition of these four ZF have been recently delineated in the crystal structure of rN-ZAP [57]. Previous studies demonstrated that rN-ZAP retains the antiviral function and the C-terminal region of rZAP is dispensable for ZAP's antiviral activity [37], [46], which is consistent with our observation that expression of rN-ZAP was sufficient to reduce HBV mRNA (Fig. 1). In order to determine if the N-terminal portion of hZAP possesses an anti-HBV function similar to the full length ZAP, the N-terminal 254 a.a. homologue of hZAP was expressed in HBV transfected HepG2 cells (Fig. 5A). The results showed that hN-ZAP reduced HBV RNA to a similar extent as both hZAP-S and rN-ZAP did (Fig. 5B), suggesting that the N-terminus of hZAP is the functional antiviral domain against HBV.

Among the four zinc-finger motifs within the N-terminus of rat ZAP, it has been reported that disruption of the second and fourth zinc fingers abolished ZAP's activity against retroviral RNA, whereas disruption of the first and third zinc fingers only slightly lowered its activity [44]. To determine the requirement of each zinc finger in hZAP-mediated HBV RNA reduction, amino acid mutation was introduced into each zinc finger motif of ZAP-S to disrupt the CCCH-type zinc finger structures individually or all together according to the previous studies [44] (Fig. 5A). The mutant ZAP-S proteins were coexpressed with HBV in HepG2 cells and their effects on viral RNA and DNA were analyzed. While disruption of each individual zinc fingers, specifically ZAP-S H86K, C88R, C168R, H191R, partially decreased the antiviral activity of hZAP-S (Fig. 5C), ablation of the entire four zinc fingers (ZAP-SΔ4ZFs) completely abolished ZAP-mediated HBV RNA decay (Fig. 5D), indicating that each zinc finger contributes to the anti-HBV function of ZAP, and the optimal antiviral activity of ZAP requires the integrity of all the four zinc fingers.

Interestingly, increased steady state levels of ZAP-S were observed when the zinc finger motifs were mutated (Fig. 5C and D, bottom panels), indicating that the zinc finger motifs may influence the stability of ZAP.

### ZAP binds to HBV RNA through zinc finger motifs

**Figure 6:**
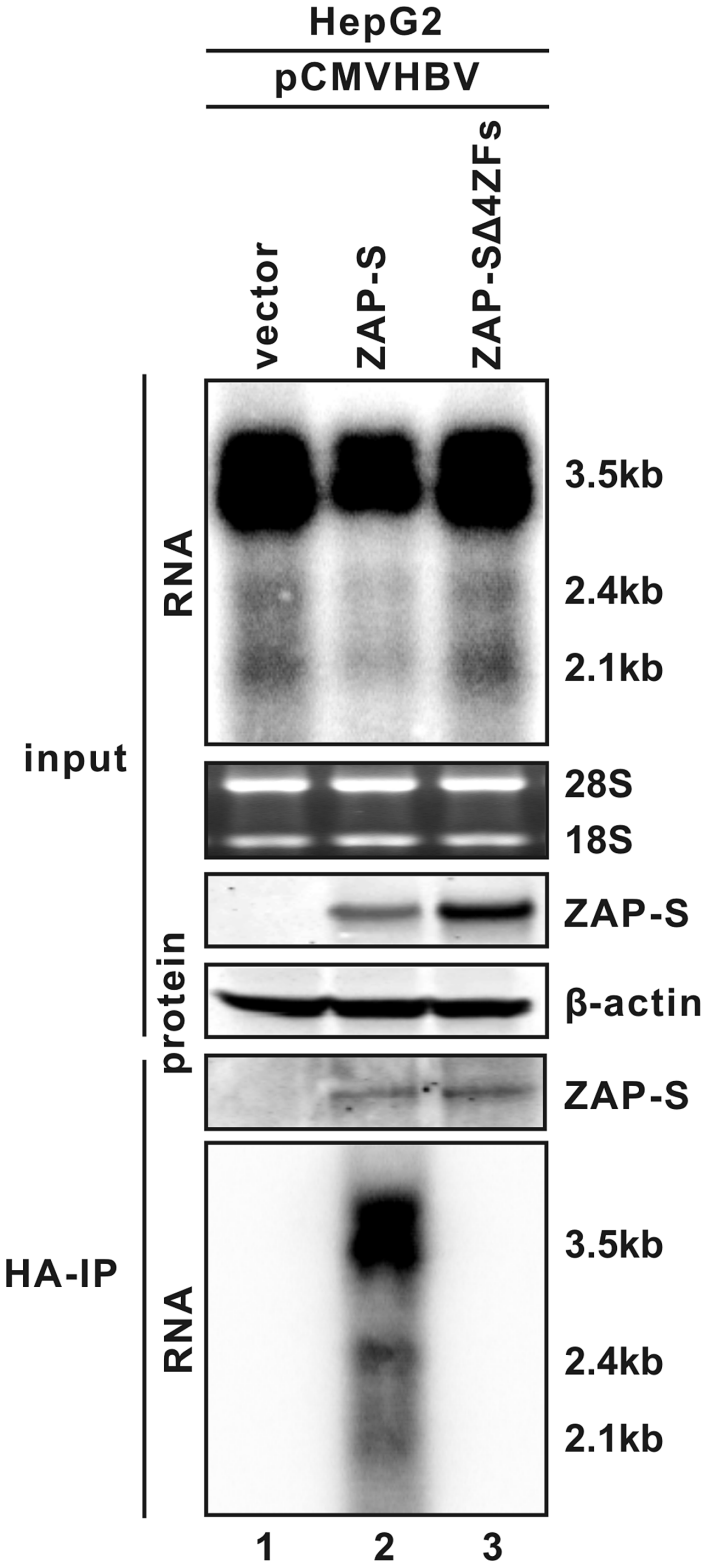
ZAP interacts with HBV RNA through its zinc finger motifs. HepG2 cells in 35 mm dishes were cotransfected with 3 µg of pCMVHBV, and 1 µg of control vector, or ZAP-S, or ZAP-SΔ4ZFs. Cells were harvested at day 3 post transfection. One set of cell samples was used to analyze HBV RNA and ZAP proteins as input controls. Another set of cells were lysed with cell lysis buffer containing RNase inhibitors. Immunoprecipitations were performed with beads covalently coated with anti-HA antibodies. Bonded HBV RNA and ZAP proteins were analyzed by Northern blot and Western blot assays, respectively (see Materials and Methods for details). Beads coated with anti-FLAG antibodies served as negative control (data not shown).

Host CCCH-type zinc finger-containing proteins have been reported to be able to bind specific RNA species [58]–[60], and rN-ZAP has been shown to be capable of binding to MMLV and Sindbis virus RNA fragments [44]. The observed zinc finger-dependent reduction of HBV RNA by hZAP suggested that the protein may interact with HBV RNA as well. To test this hypothesis, a cell based co-immunoprecipitation assay was performed. As shown in Fig. 6, with HBV expression alone serving as a negative control (lane 1), immunoprecipitation of HA-tagged wildtype ZAP-S from the lysate of HBV cotransfected HepG2 cells pulled down HBV pgRNA and subgenomic RNAs (lane 2), but HBV RNA was not co-precipitated with ZAP-SΔ4ZFs even though equivalent pull-down levels of mutant and wildtype ZAP were detected by Western blot (lane 3, compared to lane 2). Immunoprecipitation with anti-FLAG antibodies did not capture any ZAP protein and HBV RNA (data not shown). These data, in combination with the results from Fig. 5, support the notion that ZAP binds to HBV RNA through zinc finger motifs, and such protein-RNA interaction is required for the antiviral activity of ZAP against HBV.

### Mapping the ZRE sequences in HBV genome

The above observations demonstrated that HBV RNA is regulated posttranscriptionally by ZAP, and that ZAP physically interacts with HBV RNA, suggesting that ZAP targets specific HBV RNA sequences for degradation. Such RNA sequences, specifically ZAP-responsive elements (ZREs), have been previously identified in certain retrovirus, filovirus, and alphavirus RNA genomes [39], [42]–[44], [61]. However, while sequence homology exists among identified retroviral ZREs, neither sequence identities nor secondary structure similarities have been found between ZREs from different viruses [43], and HBV pgRNA does not display apparent sequence homology to any of those known ZREs (data not shown).

**Figure 7:**
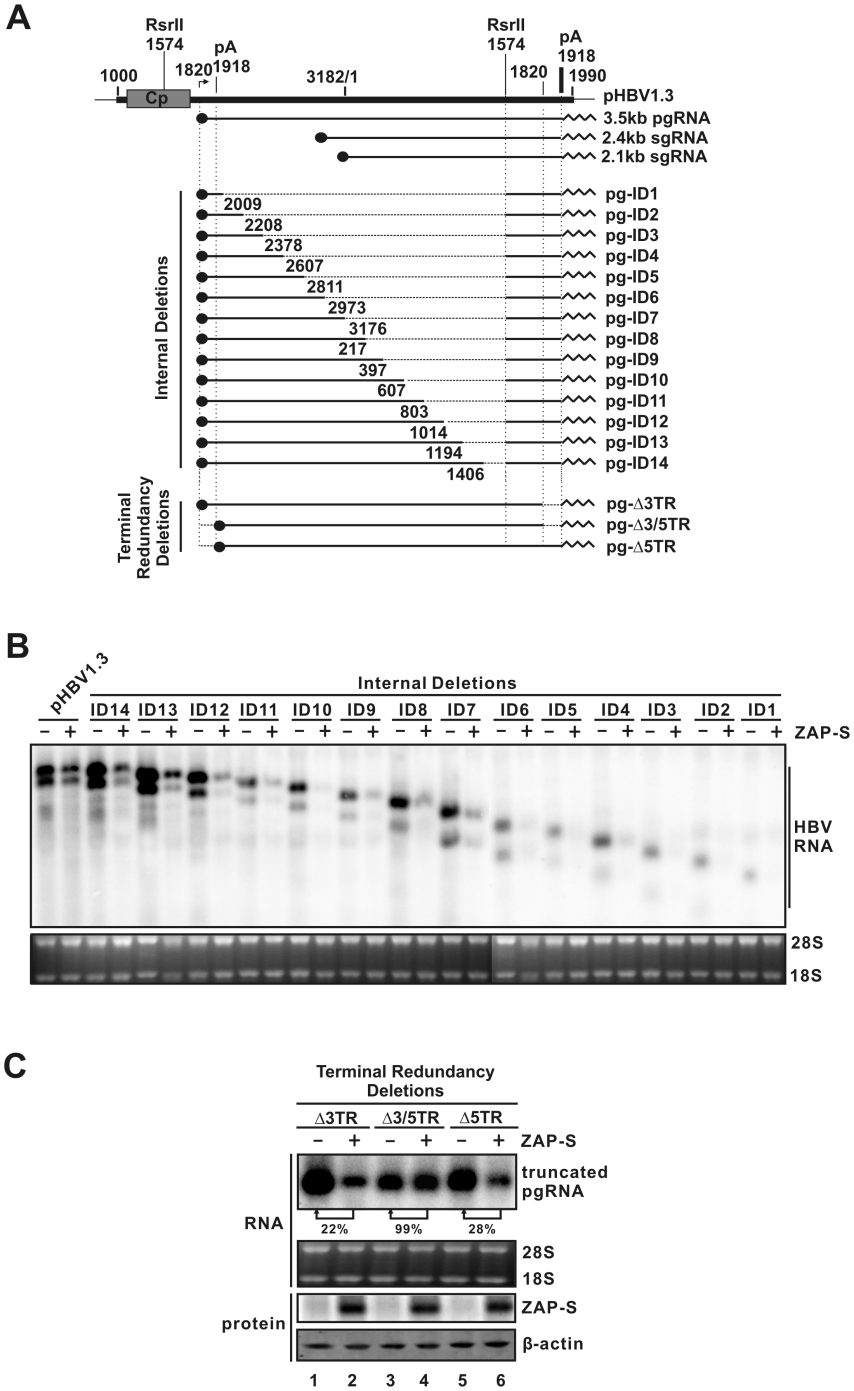
Mapping the ZRE sequences in HBV genome. (A) Schematic illustration of the construction strategy of HBV deletion clones. The plasmid pHBV1.3 contains a 1.3 overlength HBV genome, starting at nt 1000. The HBV nucleotide positions are according to Galibert et al. [83]. Cp represents the HBV core promoter. pA is the polyadenylation site. The arrow indicates the pgRNA transcription initiation site (nt 1820). Three major HBV mRNA (3.5 kb, 2.4 kb, and 2.1 kb) are depicted underneath the 1.3 mer HBV DNA template. The solid dot indicates 5′ cap of mRNA; and the sawtooth line represents the polyA tail at the 3′ terminus of mRNA. The deleted HBV sequences are drawn as broken lines. The deleted regions of the internal deletion clones (pg-ID1 to pg-ID14) are between the indicated 5′ positions and a fixed 3′ position at the second Rsr II restriction site (nt 1574). The terminal redundancy (TR) deletion clones contain truncations of HBV sequences (nt 1820–1918) at either 3′ and 5′ terminus of pgRNA coding sequences (pg-Δ3TR and pg-Δ5TR, respectively.), or both (pg-Δ3/5TR). The viral mRNA transcribed from the internal deletion clones are under the control of HBV Cp in the pHBV1.3 backbone. The transcription of terminal truncated pgRNA is governed by CMV-IE promoter in the pCDNA3.1/V5-His-TOPO vector (see Materials and Methods for detail). (B) Sensitivity of HBV pgRNA with internal sequence deletions to ZAP-mediated RNA reduction. Plasmid pHBV1.3 and the internal deletion clones were transfected into HepG2 cells individually with control plasmid or ZAP-S expression vector. Four days later, viral RNA was analyzed by Northern blot. (C) Sensitivity of HBV RNA with TR deletion to ZAP-mediated RNA decay. HepG2 cells were transfected with HBV TR deletion clone and control plasmid or ZAP-S. Cells were harvested at day 4 post transfection and subjected to viral RNA analysis by Northern hybridization (top panel). Relative level of HBV RNA under ZAP-S expression is expressed as the percentage of RNA level in the corresponding control samples, and is presented underneath the blot. The expression of HA-tagged ZAP-S was revealed by Western blot, with β-actin serving as loading control.

To detect potential HBV ZRE(s) that confer susceptibility to ZAP-mediated viral RNA degradation, we scanned the entire 3.5 kb HBV pgRNA genome. To this end, 14 internal deletion clones and 3 terminal deletion clones of the HBV genome were constructed to express HBV RNA fragments (Fig. 7A), and their sensitivities to ZAP-mediated RNA reduction were analyzed. Albeit the RNA expression levels varied among different constructs, most likely due to the absence of potential *cis*-elements maintaining HBV RNA stability, pgRNAs with consecutive internal deletions (nt 2009-3182/1-1574) remained sensitive to ZAP-S, implying that the ZRE exits in the terminal regions of HBV RNA (Fig. 7B). Interestingly, when the terminal redundancy (TR, nt 1820–1918) was removed from either the 3′ (pg-Δ3TR) or 5′ (pg-Δ5TR) terminus of pgRNA, the truncated pgRNA was still vulnerable to ZAP-S (Fig. 7C, comparing lane 2 to 1, lane 6 to 5, respectively); however, with removal of both TR from pgRNA, ZAP-S no longer reduced the level of truncated pgRNA (Fig. 7C, comparing lane 4 to3), indicating that one copy of the terminal redundant sequences is sufficient to confer the susceptibility of HBV pgRNA to ZAP-mediated degradation. Thus, the HBV ZRE maps to the TR sequences of HBV RNA.

**Figure 8:**
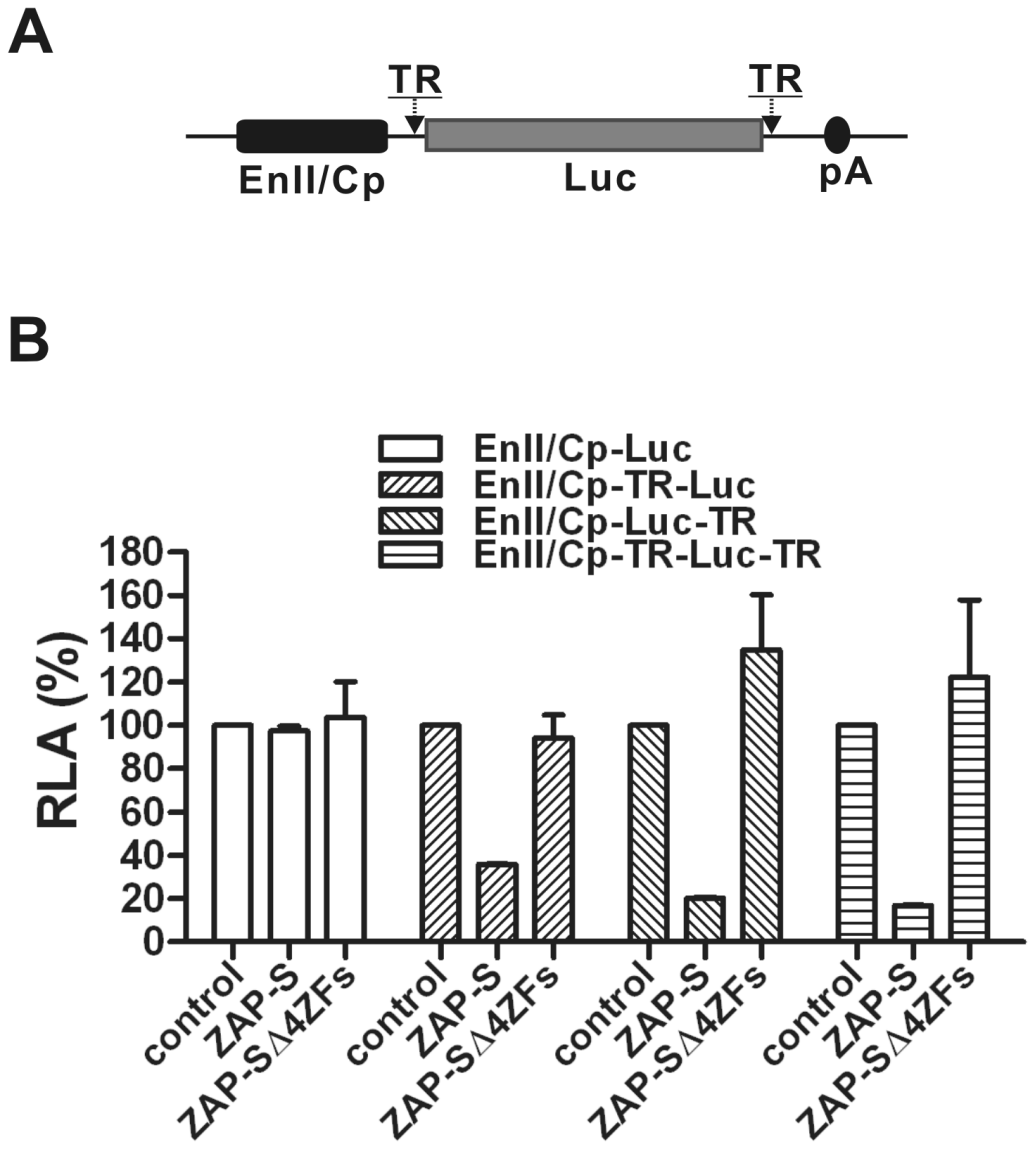
HBV TR confers the susceptibility to ZAP-mediated RNA decay. (A) Schematic structure of reporter construct EnII/Cp-Luc with HBV TR insertion at the flanking nontranslational region of luciferase ORF. See Materials and Methods for cloning details. (B) HBV TR insertion renders Luc gene to be sensitive to ZAP-S. HepG2 cells were transfected with each indicated reporter plasmid and control vector or plasmid expressing ZAP-S or ZAP-SΔ4ZFs. Cells were harvested at day 3 post transfection and luciferase activity was measured. The plotted relative luciferase activity (RLA) represents the mean ± SD (n = 4) of the percentage of absorbance obtained from wells transfected with ZAP-S or ZAP-SΔ4ZFs over control vector.

To further confirm the functionality of the HBV ZRE, the TR region was inserted into the reporter plasmid EnII/Cp-Luc, either at the upstream (EnII/Cp-TR-Luc) or downstream (EnII/Cp-Luc-TR) non-coding region of the firefly luciferease mRNA within the multiple-cloning sites, or into both flanking regions (EnII/Cp-TR-Luc-TR) (Fig. 8A). The plasmids were transfected into HepG2 cells individually, or cotransfected with plasmids expressing ZAP-S or ZAP-SΔ4ZFs, and luciferase activities were measured. As shown in Fig. 8B, insertion of HBV TR into the luciferase mRNA non-coding regions resulted in significant reduction of luciferase activity by ZAP-S expression, but not ZAP-SΔ4ZFs. Therefore, the HBV TR sequence and zinc finger motifs dependent RNA down-regulation by ZAP has been demonstrated.

### Basal and inducible expression of ZAP in hepatocytes

**Figure 9:**
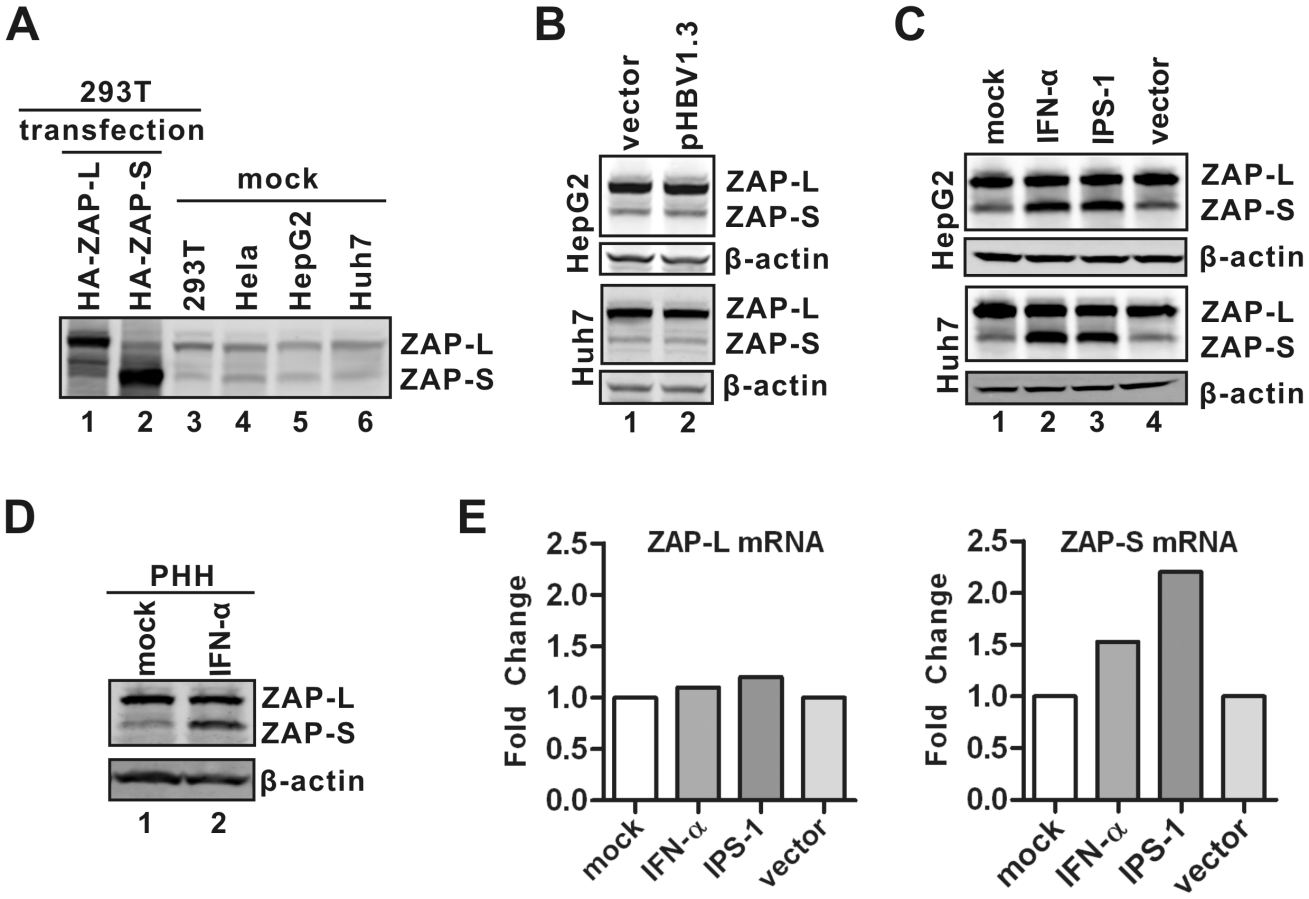
Expression of endogenous ZAP in hepatocytes. (A) The basal expression of ZAP in indicated cell lines was detected by Western blot using polyclonal antibodies against endogenous ZAP. Overexpressed HA-tagged hZAP-L and -S were used as controls to validate antibody specificity. (B) HBV replication does not affect ZAP expression. HepG2 and Huh7 cells were transfected with control vector or pHBV1.3, respectively. Expression of ZAP was analyzed by Western blot at day 4 post transfection. (C–D) ZAP expression upon IFN-α treatment or IPS-1 expression. HepG2 and Huh7 cells were left untreated or treated with IFN-α (1,000 IU/ml) for 48 h. Another set of cells was transfected with plasmid expressing IPS-1 or control vector for 48 h. Primary human hepatocytes (PHH) were treated with IFN-α (1,000 IU/ml) for 48 h. Cells were then lysed and the levels of ZAP were revealed by Western blot, with β-actin serving as loading control. (E) Quantitative RT-PCR analysis of ZAP-L (left) and ZAP-S (right) mRNA in HepG2 cells stimulated for 48 h with IFN-α (1,000 U/ml) or IPS-1 expression. The results were presented as fold change of mRNA levels compared to control samples.

It has been reported that expression of the hZAP (-L, -S) mRNA occurs in a wide variety of tissues, including both germline and somatic cells [41]. To determine the protein expression profile of hZAP in hepatocyte-derived and other established cell lines, we tested hZAP antibodies from different commercial sources and obtained one with specificity for both hZAP-L and -S detection in Western blot assay. As shown in Fig. 9, both transfected and endogenous ZAP isoforms were detected in a panel of cell lines tested in this study, including HeLa, 293T, hepatocyte-derived HepG2 and Huh7 cells (Fig. 9A), and primary human hepatocytes (PHH) (Fig. 9D). While HBV replication did not alter the expression levels of ZAP-L and -S in HepG2 and Huh7 cells (Fig. 9B), the expression of ZAP-S, but not the -L isoform, was upregulated in these two cell lines and primary human hepatocytes under IFN-α treatment or IPS-1 expression (Fig. 9C–D), suggesting that ZAP-S is an hepatic interferon stimulated gene (ISG) in response to the activation of cellular innate immunity. The preferential induction of ZAP-S by IFN-α or IPS-1 in hepatocytes is consistent with a recent report showing ZAP-S mRNA, rather than the full-length ZAP-L, was further induced by 5′-triphosphate-modified RNA in 293T cells [62]. We also found that ZAP-S mRNA was selectively induced in HepG2 cells upon IFN-α treatment or IPS-1 expression (Fig. 9E), indicating a ZAP-S mRNA-specific splicing event. The mechanisms underlying differential regulation of the splicing of ZAP mRNA precursor by innate signaling remain unclear.

Chronic hepatitis B is associated with inflammatory cytokine production upon the activation of host immunity, and the dynamic balance between host immune responses and virus replication is thought to determine the disease progression [21], [22]. It is generally acknowledged that cytokine-induced intrahepatic genes play indispensable roles in controlling HBV replication [63]–[65]. To investigate the role of ZAP in chronic HBV infection, we analyzed the levels of ZAP mRNA in liver biopsy samples obtained from chronic hepatitis B patients. According to the natural history of HBV infection, those patients were grouped into three phases (or types of immune responses), specifically immune tolerant phase, immune active phase, and inactive phase [66] (Table S1). As summarized in Fig. S4, comparing to immune tolerant and inactive carriers, patients in the immune active group have significantly elevated expression levels of both ZAP-L and -S mRNA in liver, and the upregulation of ZAP-S mRNA is more remarkable than ZAP-L. Generally, HBV patients in immune active phase exhibit episodes of hepatitis over a period of months or years as the immune system attempts to clear the infection, resulting in the decline of viral loads [67] (Table S1). Thus, we speculate that the upregulation of ZAP may play a role in immune control of HBV replication in chronic infection.

### Involvement of ZAP in IPS-1 mediated reduction of HBV RNA

Host cells are able to sense viral components through an array of pattern recognition receptors (PRRs), leading to the activation of innate cellular defense responses to combat virus infection [23]. We previously reported that activation of PRR-elicited innate immune signaling by ectopic expression of PRR-associated adaptor proteins, such as the adaptor of RIG-I-like helicases, IPS-1 (also known as MAVS/Cardif/VISA) [68]–[71], significantly inhibited HBV replication in both HepG2 and Huh7 cells [53]. The primary IPS-1-mediated antiviral effect is to reduce the levels of HBV RNA and involves posttranscriptional mechanisms of viral RNA decay [53].

**Figure 10:**
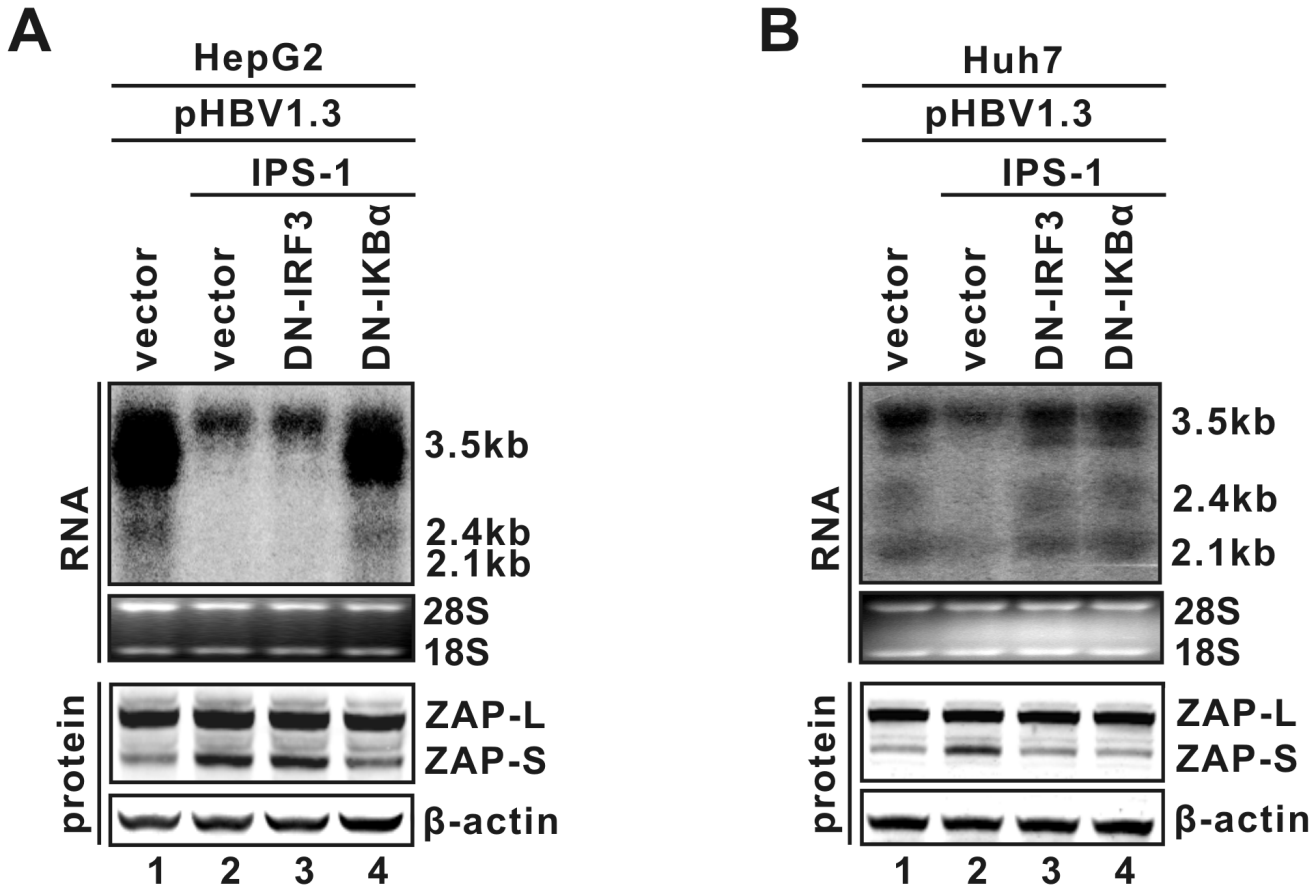
Signaling pathway dependency of IPS-1-induced HBV RNA reduction and ZAP-S upregulation in hepatocyte-derived cells. HepG2 (A) or Huh7 (B) cells in 35 mm dishes were cotransfected with 2 µg of plasmid pHBV1.3 (lanes 1–4) and 2 µg of control vector (lane 1), or 1 µg of plasmid expressing IPS-1 (lanes 2–4) plus 1 µg of control vector (lane 2) or 1 µg of plasmid expressing DN-IRF3 (lane 3) or DN-IκBα (lane 4). Cells were harvested 4 days after transfection, and the levels of viral RNA and endogenous ZAP were determined by Northern blot hybridization (upper panel) and Western blot (bottom panel), respectively.

It is well known that the RIG-I/IPS-1 complex regulates host gene expression (i.e. IFN-β) through two major signal transduction cascades, the IRF3 and NF-κB pathways (Fig. S5) [69], [72], [73]. In the previous study, we demonstrated that the inhibition of HBV replication by IPS-1 appears to be mediated by intracellular antiviral pathway(s), rather than the secretion of antiviral cytokines such as IFN-α/β. Furthermore, we found that activation of the downstream NF-κB pathway, but not the IRF3 pathway, is essential for IPS-1-mediated HBV RNA reduction in HepG2 cells; whereas activation of both pathways is required for IPS-1 to destabilize HBV RNA in Huh7 cells [53]. The differential requirements of the IRF3 and NK-κB pathways by IPS-1-mediated HBV RNA reduction in HepG2 and Huh7 cells are shown in Fig. 10 (Top panels). Considering that 1) IPS-1 induces ZAP-S expression in both cell lines (Fig. 9), 2) ZAP is capable of targeting HBV RNA for degradation (Fig. 1, 3), and 3) the sequences responsible for IPS-1-mediated antiviral response were previously mapped within the approximately 1 kb region at the 3′ terminus of HBV RNA [53], which contains HBV ZRE, we hypothesized that ZAP might be an end effector in the IPS-1-elicited anti-HBV state.

To test this hypothesis, we first analyzed the dependency on IRF3 or NF-κB signaling of IPS-1-induced ZAP expression in hepatocyte-derived cells. As shown in Fig. 10, IPS-1 expression led to an upregulation of ZAP-S in both HepG2 and Huh7 cells (Fig. 10A/B, bottom panels, comparing lane 2 to 1). While inhibition of IRF3 by dominant negative (DN)-IRF3 only blocked IPS-1-induced ZAP-S expression in Huh7 cells (Fig. 10B, bottom panels, comparing lane 3 to 2), antagonizing the NF-kB pathway by DN-IκBα attenuated IPS-1-mediated ZAP-S induction in both cell lines (Fig. 10A/B, bottom panels, comparing lane 4 to 2). Such differential dependency on IRF3 and NF-κB of IPS-1-regulated ZAP-S expression perfectly matches the cell type-dependent signaling pathway requirement for IPS-1-elicited HBV RNA reduction.

**Figure 11:**
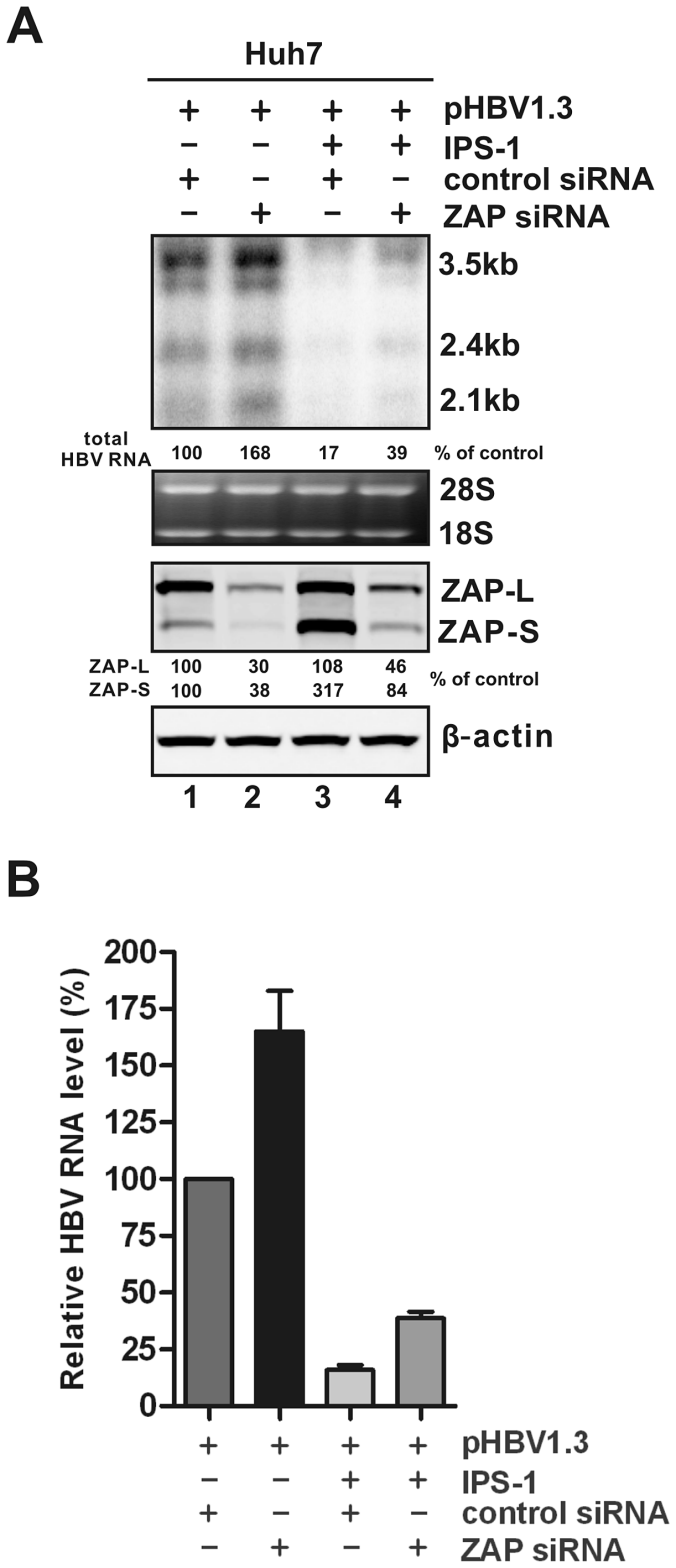
Knock down of ZAP expression increases the steady state level of HBV RNA. (A) Huh7 cells in 35 mm dishes were transfected with 50 nM of control siRNA (lanes 1 and 3) or hZAP siRNA (lanes 2 and 4). At the second day, cells were repeatedly transfected with the same siRNA at same amount used in the previous transfection, together with 1 µg of pHBV1.3 and 1 µg of control vector (lanes 1 and 2), or 1 µg of pHBV1.3 plus 1 µg of plasmid expressing IPS-1 (lanes 3 and 4). Cells were incubated for an additional 48 h and harvested for HBV RNA and ZAP protein analyses by Northern and Western blot, respectively. Relative level of HBV RNA or ZAP isoforms in each sample is expressed as the percentage of RNA or protein level in the control sample (lanes 1), and is presented underneath each of the blots. (B) Viral RNA levels were quantified from three experimental trials and plotted as relative level of control samples (mean ± SD).

We then attempted to knock down the cellular expression level of ZAP. The Huh7 line was selected for the knock down experiments because higher sequential transfection efficiency can be achieved in comparison to HepG2 cells. As shown in Fig. 11, ZAP-specific siRNA reduced the basal levels of both ZAP-L and -S by more than 50%, together with a significant increase of HBV RNA expression (Fig. 11A, comparing lane 2 to 1; Fig. 11B), suggesting that the basal level of ZAP is a robust host restriction factor for HBV. However, knock down of ZAP only partially attenuated the antiviral effect of IPS-1, indicating that while ZAP plays a role in IPS-1-elicted HBV RNA reduction, additional IPS-1-induced host end-effectors with similar or distinct antiviral mechanisms against HBV RNA likely are required for IPS-1 to exert maximal antiviral activity.

## Discussion

Serving as an intrinsic host antiviral factor, ZAP has been reported to inhibit the infection of a variety of RNA viruses, including MMLV, HIV-1, XMRV, certain alphaviruses and filoviruses [37]–[39], [42], [43]. ZAP was initially identified in rat, and the conserved homologues have since been cloned from mouse, human and other primates [37], [41], [62]. The cross-species expression and broad tissue distribution of ZAP indicate that it has been evolutionarily preserved in mammals, suggesting a long history of active antiviral activities in host-pathogen interactions. However, ZAP is not a universal antiviral factor, as HSV-1 and yellow fever virus are resistant [38]. It had been speculated that ZAP exclusively antagonizes certain RNA viruses, until a recent report demonstrating that ZAP regulates murine gammaherpesvirus 68 latent infection by reducing viral M2 mRNA [47]. In this report, we demonstrated that ZAP inhibits the replication of HBV, a DNA pararetrovirus belonging to *Hepadnaviridae* family, primarily through down-regulation of viral mRNA. Our findings thus further expand the antiviral spectrum of ZAP and provide more information for a better understanding of ZAP biology.

ZAP mediates an antiviral function through direct binding to viral RNA, resulting in RNA degradation. The RNA binding activity of ZAP is managed by the N-terminal portion of the protein, which contains four tandem CCCH-type zinc finger motifs [44]. Interestingly, the antiviral activity of rat ZAP was first discovered using a truncated ZAP protein (r-NZAP), which consists only of the four zinc fingers that mediate RNA binding [37]. The human ortholog of ZAP (hZAP) gene encodes two protein isoforms that result from alternative splicing of a C-terminal poly(ADP-ribose) polymerase (PARP)-like domain, which is present in the longer ZAP isoform (hZAP-L), but not the shorter version (hZAP-S). However, both hZAP-L and -S have been shown to inhibit MMLV, Semliki Forest virus, HIV-1 [41], [42], and HBV (this report), suggesting that the PARP domain is not essential to support the antiviral activity of ZAP. While the overall protein sequence homology between rZAP and hZAP is about 50%, their N-terminal portion of 254 a.a. share approximately 80% identity, and the four zinc-finger motifs are highly conserved [41], [56]. In addition, we demonstrated that both rN-ZAP and its human 254 a.a. homolog (hN-ZAP) are able to reduce HBV RNA, which further confirmed that the N-terminus of ZAP is the functional antiviral domain.

Among the four zinc finger motifs in the N-terminal portion of ZAP, the 2^nd^ and 4^th^ zinc fingers plays the major role in inhibition of MMLV by rat ZAP [44]. For HBV, we found that each of the four zinc fingers partially and equally contributes to hZAP-mediated viral RNA decay, and the integrity of all four zinc fingers is required for ZAP to achieve a maximal inhibition of HBV (Fig. 5). In addition, we demonstrated that ZAP is able to interact with HBV RNA in a co-immunoprecipitation assay, and disruption of the full set of zinc finger motifs completely abolished the binding of ZAP to HBV RNA (Fig. 6). Recently, the crystal structure of rN-ZAP has been solved [57]. The overall structure of rN-ZAP molecule resembles a “tractor-like” shape, with the four zinc finger motifs forming a large putative RNA-docking cleft comprised of two cavities. Further structural and functional analysis revealed that multiple positively-charged residues scattered in the CCCH-type zinc finger motifs also coordinately contribute to the formation of RNA-binding grooves. The structural complexity of the RNA-binding domain suggests that ZAP may bind to different target RNA slightly differently, and thus the contribution and requirement of each zinc finger motif may vary with the binding of different RNA substrates [57].

Previous mutagenesis and structure studies suggested that ZAP forms a homodimer for its RNA binding and antiviral activities, and the ZAP self-interacting domain largely overlaps with its antiviral zinc finger motifs [57], [74]. In this report, we demonstrated that the nonfunctional hZAP mutant (ZAP-SΔ4ZFs) failed to bind HBV RNA (Fig. 6), and did not exhibit dominant-negative effect on endogenous or overexpressed wild-type ZAP (Fig. 5D, Fig. S6), favoring the notion that the zinc finger-mediated ZAP self-association is required for ZAP to directly target RNA for degradation.

ZAP binds to specific viral RNA through the recognition of a so-called ZAP responsive element (ZRE) [44], [46]. Among the ZREs previously mapped in viral RNA genomes, including MMLV, HIV-1, XMRV, and certain alphaviruses and filoviruses, no obvious common RNA sequences or structural motifs could be identified, although they are all more than 500 nt long [39], [42]–[44], [46]. Herein, we mapped the HBV ZRE to the 100 nt terminal redundancy region (nt 1820–1918) of HBV mRNA, which is present at both termini of viral pgRNA and at the 3′ end of viral subgenomic RNAs. The presence of a ZRE in the HBV genome further confirms that ZAP- mediated HBV RNA reduction is due to posttranscriptional regulatory mechanisms, similar to the reported antiviral mechanism of ZAP against other viruses. However, alignment of HBV ZRE with other individual viral ZREs did not reveal any obvious sequence homology (data not shown), suggesting that a mysterious secondary or tertiary RNA structure may define the common feature of ZRE. A recent SELEX (Systematic Evolution of Ligands by Exponential Enrichment) study showed that ZAP-binding aptamers that were 40-nt-long G-rich RNAs with stem-loop structures containing conserved “GGGUGG” and “GAGGG” motifs in the loop region [75]. Interestingly, HBV ZRE contains a stem-loop region (epsilon (ε), nt 1849–1909) which serves as the pgRNA encapsidation signal and as the priming template for virus reverse transcription [6], [7], [76]. A sequence of “GGGUGG” (nt 1888–1893) also exists, but, in the upper stem region of the epsilon (Fig. S7). The HBV TR structure and sequence partially match the proposed criteria of ZRE from the SELEX study. Our preliminary data showed that insertion of ε sequence alone at the 3′ nontranslationed region of the luciferase gene resulted in a significant reduction of luciferase activity when coexpressed with ZAP (data not shown). Future investigations on HBV ZRE will focus on determining the minimal sequence or structural requirements and the key residues for a functional ZRE.

Despite acting as a host antiviral factor, the other potential functions of ZAP in regulating host cellular function remain largely elusive. Since ZAP targets viral mRNA for destabilization, it is conceivable that ZAP may also alter the stability of certain cellular mRNA to regulate host gene expression. This notion is supported by previous observations that the mRNA level of the B3GALTL gene was significantly reduced by rNZAP overexpression in rat fibroblast cells [46]. In addition, considering ZAP favors short RNAs with stem-loop structures for affinity binding, it is also possible that ZAP potentially targets host microRNA (miRNA) precursors, including pri-miRNA and pre-miRNA with about 70 nt hairpin loop structures [77], to regulate miRNA processing and maturation. This hypothesis can be investigated by host target prediction using homology searches with the identified viral ZRE sequences, or through microarray analysis of gene profiling after ZAP overexpression or knock down.

A previous study reported that hZAP-S associates with RIG-I to stimulate IFN-β production through activation of IRF3 and NF-κB pathways in cell culture, suggesting hZAP-S is able to activate host innate defense machinery for virus inhibition [62]. To explore whether RIG-I signaling is involved in ZAP-mediated HBV RNA decay, we found that overexpression of ZAP-S had little ability to stimulate the IFN-β promoter activity in HepG2 cells (Fig. S8A). Inhibition of IRF3 and NF-κB signaling did not restore the levels of HBV RNA under ZAP-S overexpression (Fig. S8B), suggesting that ZAP-mediated HBV RNA reduction is not largely due to the stimulation of RIG-I pathway, which is consistent with a recent observation that knock down of RIG-I did not abrogate the inhibitory effect on XMRV by ZAP [43]. In contrast, ZAP-S expression can be further induced by activation of the RIG-I pathway through IPS-1 expression, and knock down of ZAP partially attenuated the antiviral activity of IPS-1, thus indicating that ZAP is one of the contributing end-effectors in the innate control of HBV replication (Figs. 9–11). In addition, we observed that ZAP-S protein expression was further induced in primary human hepatocytes by IFN-α treatment (Fig. 9D), and the levels of ZAP mRNA were elevated in the livers of hepatitis B patients during the immune active phase (Fig. S4), implying that ZAP may also play a role in host immune responses against HBV infection *in vivo*.

ZAP does not encode any ribonuclease activity [46]. It has been reported that rat ZAP binds to the ZRE of MMLV RNA and recruits the host RNA processing exosome to degrade the viral RNA substrate from its 3′ end [45]. It is of interest to determine whether the exosome is involved in ZAP-mediated HBV RNA decay. However, our attempts to knock down the exosome subunits, such as EXOSC4, have not been successful in hepatocyte-derived cells. Alternatively, a chemical inhibitor, Fluorouracil (5-FU), was employed to inhibit exosome activity in our study. The results showed that 5-FU treatment partially attenuated the viral RNA reduction under ZAP-S overexpression or IFN-α treatment, but only marginally increased the level of HBV RNA in the control group (Fig. S9). Although a possibility that the attenuated antiviral effect by exosome inhibition was due to a ZAP-independent mechanism could not be completely ruled out, the obvious antiviral attenuation by 5-FU under ZAP-S overexpression and IFN-α induction implied that the exosome activity might be required for ZAP-mediated HBV RNA decay. In fact, previous studies demonstrated that other host enzymes are recruited by ZAP to potentiate retroviral RNA degradation, including RNA helicase p72 and DHX30 for restructuring RNA, polyA ribonuclease (PARN) for trimming the polyA tail of mRNA, and cellular decapping complex for initiating the degradation of the target viral mRNA from the 5′ end [42], [46], [78], [79]. Interestingly, HBV ZRE localizes in close proximity to both termini of pgRNA and to the 3′ end of subgenomic RNA, which it may efficiently direct ZAP and its partners to the RNA degradation initiation sites. Whether those co-factors are involved in ZAP-mediated HBV RNA degradation in heptocytes awaits further investigation.

It also has been reported that ZAP works in concert with other ISGs to confer maximal protection against virus infection upon IFN treatment [40], [49]. Rat ZAP-synergistic ISGs for alphavirus inhibition have been identified recently [61]. In our previous work on screening of antiviral ISGs for HBV inhibition, a handful of ISGs that reduce the steady state level of HBV RNA have been identified [31]. Among them, interferon-stimulated gene 20 kDa protein (ISG20) is an exoribonuclease that down-regulates HBV RNA in a posttranscriptional fashion similar to ZAP (Nie and Guo, unpublished data). The detailed molecular mechanisms of ISG20-mediated HBV RNA decay, and its potential synergistic activity on ZAP's anti-HBV function, are currently under way.

Taken together, the work reported herein has identified ZAP as a host restriction factor that limits HBV replication. Mechanistic studies of the inhibitory effect of ZAP on HBV replication have important implications for a better understanding of virus-host interaction, host antiviral immunity, and viral pathogenesis during HBV infection. Considering the importance of HBV RNA in the virus life cycle, degradation of viral RNA will result in a dramatic suppression of viral DNA replication and antigen production. Therefore, ZAP-mediated HBV RNA decay can be potentially utilized to develop novel antiviral strategies against HBV through gene delivery of ZAP expression vectors into HBV infected cells. It is also envisaged that small molecules could be discovered to mimic the antiviral function of ZAP, or provoke its antiviral activity, to achieve a beneficial therapeutic value for the management of chronic hepatitis B.

## Materials and Methods

### Ethics statement

All human subjects were recruited with informed written consent. The study was approved by the Institutional Ethics Committee for human studies at Huashan Hospital, Fudan University. All clinical investigations have been conducted according to the principles expressed in the Declaration of Helsinki.

### Gene Chip analysis of intrahepatic gene expression in patients with chronic hepatitis B

Liver biopsies were obtained from 24 hepatitis B patients enrolled in this study (Table S1). Total mRNA was isolated from liver specimens and the gene expression profiling was performed by using Affymetrix Human U133 Plus 2 arrays at Ebioservice, Inc (Shanghai, China). Microarray data were analyzed using GeneSpring GX 10 (Agilent). The complete microarray dataset will be published separately. In this report, the ZAP gene (ZAP-L, -S) expression data are expressed as log_2_ values with mean ± SD using Graphpad prism 5.0 software, a *p*-value<0.05 (t-test) is considered significant.

### Cell cultures and drugs

Human hepatocyte-derived HepG2 and Huh7 cells and human embryonic kidney 293T cells were obtained from the ATCC and maintained in DMEM/F12 medium (Mediatech) supplemented with 10% fetal bovine serum, 100 U/ml penicillin and 100 µg/ml streptomycin. HepDES19 cells were maintained in the same way as HepG2, but with the addition of 1 µg/ml tetracycline and 400 µg/ml G418 [80]. To initiate HBV replication in HepDES19 cells, tetracycline was withdrawn from the culture medium and the cells were cultured for the indicated time. Cryopreserved primary human hepatocytes were obtained from Triangle Research Labs, LLC (Research Triangle Park, NC); cells were recovered and cultured according to supplier's protocol. Recombinant human IFN-α2a was purchased from PBL Biomedical Laboratories. 5-fluorouracil (5-FU), an exosome inhibitor, was purchased from Sigma-Aldrich and dissolved in DMSO (10 µg/ml stock).

### Plasmids

HBV (genotype D, subtype ayw, Genbank accession number V01460.1) replication competent plasmids, pHBV1.3 and pCMVHBV, in which the transcription of viral pgRNA is governed by authentic HBV core promoter and CMV-IE promoter, respectively, were described previously [31], [53], [81]. HBV pgRNA internal deletion clones (pgID-1 to pgID14) were constructed on the pHBV1.3 backbone by PCR. Plasmids expressing the terminal redundancy (TR, nt 1820–1918) truncated viral pgRNA were obtained by insertion of TR-absent (either 5′ or 3′ TR, or both) HBV pgRNA coding sequence into pCDNA3.1/V5-His-TOPO vector (Life Technologies) (refer to Fig. 7A for schematic illustration). Plasmids pE and pS that encode HBV 2.4 kb and 2.1 kb mRNA for the expression of HBV envelope proteins were kindly provided by Dr. Volker Bruss (Institute of Virology, Helmholtz Zentrum München, Germany) [82]. To construct an HBV core promoter (Cp) reporter plasmid, a fragment covering the HBV enhancer II (EnII) and Cp region (nt 1400–1820) was PCR amplified from plasmid pHBV1.3 and inserted into the SacI and HindIII restriction sites in pGL3-Basic vector (Promega). The generated plasmid was designated EnII/Cp-Luc, in which expression of the firefly luciferase reporter gene is governed by the HBV core promoter. Reporter plasmid EnII/Cp-TR-Luc was constructed by inserting an HBV DNA fragment spanning the viral EnII/Cp region and 5′ TR (nt 1390–1932) into the SacI/HindIII restriction sites in pGL3-Basic vector. Reporter plasmid EnII/Cp-Luc-TR bears TR-containing sequence insertion (nt 1804–1930) at the XbaI/FseI site in plasmid EnII/Cp-Luc. Similarly, XbaI/FseI restricted TR-containing fragment (nt 1804–1930) was implanted into the same restriction site in EnII/Cp-TR-Luc to generate EnII/Cp-TR-Luc-TR. HBV surface promoter reporter plasmids, namely S1-Luc and S2-Luc, were engineered through insertion of HBV DNA fragments containing S1 (nt 2708–2809) and S2 (nt 2906–3160) promoter region into the SacI and HindIII restriction sites in pGL3-Basic, respectively. CMV-IE promoter *Renilla* luciferase reporter plasmid pRL-CMV was purchased from Promega. pcDNA4-derived plasmids expressing the N-terminal 254 a.a. zinc-finger motif domain of rat ZAP (rN-ZAP) and full-length human ZAP isomers (hZAP-L, hZAP-S) were described previously and kindly provided by Dr. Malik Harmit (Fred Hutchinson Cancer Research Center) [41]. The N-terminal 254 a.a. coding region of hZAP was PCR amplified and cloned into pcDNA4 (Life Technologies) to generate plasmid hN-ZAP. Disruption of the individual or all the four putative zinc finger motifs in hZAP-S was carried out according to the previous report by using QuikChange II Site-Directed Mutagenesis Kit (Agilent) [44]. All of the above wildtype and mutant ZAP genes have an N-terminal HA-tag sequence in the expression vectors. The plasmids expressing the dominant-negative IRF3 (DN-IRF3) and dominant negative IκB-alpha (DN-IκBα) were described previously [53]. IFN-β1 promoter luciferase reporter plasmid (IFN-β1-Luc) was a gift from Dr. Hong-Bing Shu (Wuhan University).

### Cell transfection

Cells (∼1.2×10^6^) were seeded in a collagen coated 35-mm-diameter dish in antibiotics-free DMEM/F12 medium. After 6 hours, each well was transfected with a total of 4 µg plasmids with Lipofectamine 2000 (Life Technologies) by following the manufacturer's directions. Transfected cells were harvested at the indicated time points.

### Reporter assay

The HepG2 cells (∼1×10^4^) were transfected with promoter reporter plasmid plus vectors expressing gene of interest using Lipofectamine 2000. For each transfection, empty control plasmid was added to ensure that each transfection receives the same amount of total DNA (200 ng). To normalize for transfection efficiency, 4 ng of pRL-CMV *Renilla* luciferase reporter plasmid was added to each transfection. Three days after transfection, cells were harvested and lysed. Luciferase activities in cell lysates were assayed using a dual luciferase assay system (Promega) and measured by TopCount NXT. Relative firefly luciferase activities were normalized based on *Renilla* luciferase activities.

### Viral nucleic acid analysis

Intracellular HBV core DNA and total cellular RNA were extracted as described previously [53], [80]. For Core DNA analysis, one third of the DNA sample from each plate was resolved by electrophoresis into a 1.5% agarose gel and blotted onto Hybond-XL membrane (GE Healthcare). For RNA Northern blot analysis, ten microgram of total cellular RNA was resolved in 1.5% agarose gel containing 2.2 M formaldehyde and transferred onto Hybond-XL membrane. Membranes were probed with either α-^32^P-UTP (800 Ci/mmol, Perkin Elmer) labeled minus or plus strand specific full-length HBV riboprobe and exposed to a phosphorimager screen. Hybridization signals were quantified with QuantityOne software (Bio-Rad).

### Western blot assay

Cells in 35 mm dish were washed once with PBS buffer and lysed in 300 µl of 1× Laemmli buffer. Thirty microliters of the cell lysate was resolved on a 10% SDS-PAGE and proteins were transferred onto Immobilon PVDF-FL membrane (Millipore). The membranes were blocked with Western Breeze blocking buffer (Life Technologies) and probed with antibodies against HA-tag (Covance, clone 16B12), hZAP (Proteintech Group, Inc), EXOSC4 (Santa Cruz) or β-actin (Millipore). Bound antibodies were revealed by IRDye secondary antibodies. The immunoblot signals were visualized and quantified with the Li-COR Odyssey system.

### Cell fractionation

For protein analysis, the cytoplasmic and nuclear fractions of HepG2 cells were separated using the Qproteome Cell Compartment Kit (QIAgen) by following the manufacturer's directions. The purity of cytoplasmic and nuclear fractions was confirmed by measuring cytoplasmic and nuclear specific protein markers (Annexin I and Lamin A/C, respectively) with Western blot assay by following the manufacturer's procedures. For RNA analysis, cytoplasmic and nuclear RNA were isolated and purified by Cytoplasmic & Nuclear RNA Purification Kit (Norgen). RNA samples were subjected to Northern blot assay for HBV RNA detection as described above.

### Immunofluorescence

HepG2 cells were transfected with ZAP-S for 48 h and followed by fixation with 2% paraformaldehyde and permeablization of the cell membrane with 0.1% Triton X-100. Cells were then immunostained with anti-HA antibodies (Covance, clone 16B12) and the bound antibodies were visualized by Alexa Fluor 488 goat anti-mouse IgG (Life Technologies). Nuclei were counterstained with DAPI. Cells were imaged with a Nikon fluorescent microscope and photographed with a charge-coupled device camera.

### ZAP and HBV RNA co-immunoprecipitation

HepG2 cells were cotransfected with pCMVHBV and control vector, or HA-tagged wildtype or mutant ZAP-S. In order to obtain easily detectable levels of HBV RNA and ZAP, the ratio between input HBV plasmid and ZAP expression vector was optimized to 3∶1, and the transfection was maintained for 3 days. The harvested cells were lysed on ice with cell lysis buffer containing 1% NP-40, 10 mM Tris.HCl (pH 7.5), 1 mM EDTA, 50 mM NaCl, 8% sucrose, and 1 U/µl of RNasin Plus RNase Inhibitor (Promega). After centrifugation to remove the cell debris, the clarified cell lysates were incubated with EZview Red Anti-HA or Anti-FLAG Affinity Gel (Sigma-Aldrich) at 4°C for 2 h with gentle rotation. The beads were spun down and resuspended with rinse buffer (10 mM Tris.HCl (pH 7.5), 1 mM EDTA, 50 mM NaCl, 10 µM ZnCl_2_, and 1 U/µl of RNasin Plus RNase Inhibitor) for three times at 4°C. The pelleted beads were subjected to RNA extraction with TRIzol, and protein sample preparation with Laemmli buffer. Immunoprecipitated ZAP protein and HBV RNA were analyzed by Western blot and Northern blot assays, respectively.

### Quantitative RT-PCR

DNase I-treated total cellular RNA was used to generate cDNA by SuperScript III Reverse Transcriptase (Life Technologies). Real-time PCR was performed with SYBR Green Master (Roche) and the LightCycler 480 System (Roche) by using ZAP-L and -S specific primers [62]. The gene expression data were normalized to GAPDH from the same samples.

### RNAi

Control siRNA (Cat. sc-37007) and hZAP siRNA (Cat. sc-89362) were purchased from Santa Cruz Biotechnology, Inc. ZAP siRNA is a pool of 3 target-specific 19–25 nt siRNAs designed to knock down the expression of both ZAP isoforms. siRNA transfection was performed by Lipofectamine 2000 according to manufacturer's directions.

### NCBI accession numbers

HBV genomic DNA (genotype D, subtype ayw): V01460.1; human ZAP-L: NM_020119.3; human ZAP-S: NM_024625.3; rat ZAP: AF521008.1; IPS-1: NM_020746.4.

## Supporting Information

Figure S1 **Expression of ZAP reduces the steady state levels of HBV RNA in Huh7 and 293T cells.** (A) Human hepatoma Huh7 cells in 35 mm dishes were cotransfected with 2 µg of plasmid pHBV1.3 plus equal amounts of either control vector, or plasmid expressing HA-tagged hZAP-L, hZAP-S, and rN-ZAP, respectively. Cells were harvested at day 4 post transfection and viral RNAs were analyzed by Northern blot assay (top panel). Expression of ZAP was revealed by Western blot analysis with HA antibodies (bottom panel). The levels of β-actin were probed simultaneously on the same blot as loading controls. (B) Human embryonic kidney 293T cells were transfected and analyzed in the same way as Huh7 cells, except for the plasmid pHBV1.3 was replaced by pCMVHBV to bypass the requirement of liver specific transcription factors for HBV pgRNA transcription.
(TIF)

Figure S2 **ZAP expression reduces the levels of HBV subgenomic RNA.** HepG2 cells in 35 mm dishes were cotransfected with plasmids pE and pS (1 µg of each), plus 2 µg of control vector, or plasmid ZAP-S. Four days later, HBV subgenomic RNA (2.4 kb and 2.1 kb in length) were detected by Northern blot hybridization. Results from duplicate experiments are presented.
(TIF)

Figure S3 **ZAP does not reduce the levels of HBV RNA transcription template.** (A) ZAP does not promote the elimination of transfected HBV plasmid DNA. HepG2 cells in 35 mm dishes were cotransfected with 2 µg of plasmid pCMVHBV and 2 µg of control vector or plasmid that expresses ZAP-S. The cells were harvested at day 5 post transfection and viral DNA was analyzed by hybridization. During cytoplasmic HBV DNA extraction, DNase I digestion of input HBV plasmid DNA in cell lysates was omitted prior to SDS/pronase treatment, and the DNA samples were incubated with DpnI restriction enzyme to digest the bacteria-derived plasmid DNA at methylated DpnI cleavage sites. The DpnI-restricted HBV plasmid DNA fragments that migrated underneath the viral single strand DNA were revealed by electrophoresis and Southern hybridization. Expression of ZAP-S was detected by Western blot with antibodies against HA-tag. β-actin served as loading control. (B) Expression of ZAP reduces HBV RNA and DNA in stably transfected HBV cell line. Tetracycline inducible (tet-off) HBV stable cell line, HepDES19 cells, were cultured in 35 mm dish, followed by tet withdrawal and transfection of 4 µg of control vector or plasmid ZAP-S. Five days later, viral nucleic acids and ZAP-S expression were analyzed.
(TIF)

Figure S4 **Intrahepatic mRNA levels of ZAP in chronic hepatitis B patients.** Twenty-four patients were divided into three groups, specifically immune tolerant phase (8 patients), immune active phase (8 patients), and inactive carrier phase (8 patients) (Table S1). The mRNA levels of ZAP-L (A) and ZAP-S (B) from individual liver biopsy samples in each group (X-axis) were measured by gene chip analysis and plotted as log_2_ value with mean ± SD (Y-axis). *p*<0.05 is considered as significant.
(TIF)

Figure S5 **Expression of IPS-1 stimulates IFN-β promoter activity through IRF3 and NF-κB pathways.** HepG2 cells were transfected with IFN-β1 promoter luciferase reporter plasmid IFN-β1-Luc and indicated plasmid expressing IPS-1, DN-IRF3, and DN-IκBα. Luciferase assay was performed at 48 h post transfection. Relative luciferase activity was expressed as fold induction (mean ± SD, n = 3) versus control.
(TIF)

Figure S6 **ZAP-SΔ4ZFs does not possess dominant negative effect on ZAP's antiviral activity.** HepG2 cells were cotransfected with indicated plasmids for 5 days, followed by analyses of viral RNA, DNA, and ZAP-S expression.
(TIF)

Figure S7 **RNA sequences and predicted secondary structure of HBV ZRE.** HBV terminal redundancy ranges from the initiation site (nt 1820) of pgRNA to the polyadenylation site (pA, nt 1918). Stem-loop structure (epsilon, ε) is illustrated according to the literature [7]. “GGGUGG” motif is highlighted in red.
(TIF)

Figure S8 **ZAP-mediated HBV RNA reduction is independent of IFN.** (A) Overexpression of ZAP only slightly stimulates IFN-β promoter activity. HepG2 cells were transfected with indicated plasmids for 48 h. IFN-β1 promoter-driven luciferase activity was expressed as fold induction (mean ± SD, n = 3) over control. (B) Inhibition of IFN production does not rescue ZAP-mediated HBV RNA reduction. HepG2 cells in 35 mm dishes were transfected with 1 µg of each indicated plasmids, and control vector was supplemented to ensure that each transfection received the same amount (4 µg) of total transfected DNA. Cells were harvested at day 3 post transfection, HBV RNA and HA-tagged ZAP-S were analyzed by Northern hybridization and Western blot, respectively.
(TIF)

Figure S9 **Exosome activity might be involved in ZAP-mediated HBV RNA reduction.** HepG2 cells in 35 mm dishes were cotransfected with 2 µg of pHBV1.3 (lanes 1–6), and 2 µg of control vector (lanes 1, 2, 5, 6) or 2 µg of plasmid expressing HA-tagged ZAP-S (lanes 3 and 4). Twelve hours after transfection, cells were treated with 0.1% solvent DMSO (lanes 1, 3, 5), or 10 ng/ml 5-FU (lanes 2, 4, 6), two sets of pHBV1.3 and vector cotransfected cells were further treated with IFN-α (1,000 IU/ml) (lanes 5 and 6). The treatments were repeated every day for 3 days. Northern hybridization was performed to analyze HBV RNA (upper panel), and relative level of HBV RNA in each sample is expressed as the percentage of RNA level in the control samples (lanes 1), and is presented underneath the blot. The levels of ZAP and exosome subunit EXOSC4 were detected by Western blot, with β-actin serving as loading control.
(TIF)

Table S1 **The clinical characteristics of studied subjects.** A total of 24 treatment-naïve chronic hepatitis B patients were classified into 3 phases of infection according to their serum ALT and HBV DNA levels, including immune tolerant phase (8 patients; normal ALT, HBV DNA>10^5^ copies/ml), immune active phase (8 patients; elevated ALT, HBV DNA>10^5^ copies/ml), and inactive phase (8 patients; normal ALT, HBV DNA<10^4^ copies/ml). In each group, values are expressed as average (low-high) or number. Alanine transaminase (ALT); Hepatitis B surface antigen (HBsAg); Hepatitis B e antigen (HBeAg); Hepatitis B core antibody (Anti-HBc).
(DOC)

## References

[1] Liang TJ (2009) Hepatitis B: the virus and disease. Hepatology 49: S13–21. 19399811 10.1002/hep.22881 PMC2809016

[2] Block TM, Guo H, Guo JT (2007) Molecular virology of hepatitis B virus for clinicians. Clin Liver Dis 11: 685–vii, 685-706, vii. 17981225 10.1016/j.cld.2007.08.002 PMC2144742

[3] McMahon BJ (2005) Epidemiology and natural history of hepatitis B. Semin Liver Dis 25 Suppl 1: 3–8. 16103976 10.1055/s-2005-915644

[4] Dandri M, Locarnini S (2012) New insight in the pathobiology of hepatitis B virus infection. Gut 61 Suppl 1: i6–17. 22504921 10.1136/gutjnl-2012-302056

[5] Kuo A, Gish R (2012) Chronic hepatitis B infection. Clin Liver Dis 16: 347–369. 22541703 10.1016/j.cld.2012.03.003

[6] Seeger C, Mason WS (2000) Hepatitis B virus biology. Microbiol Mol Biol Rev 64: 51–68. 10704474 10.1128/mmbr.64.1.51-68.2000 PMC98986

[7] Nassal M (2008) Hepatitis B viruses: reverse transcription a different way. Virus Res 134: 235–249. 18339439 10.1016/j.virusres.2007.12.024

[8] Wolf D, Goff SP (2008) Host restriction factors blocking retroviral replication. Annu Rev Genet 42: 143–163. 18624631 10.1146/annurev.genet.42.110807.091704 PMC3598625

[9] Kirzinger MW, Stavrinides J (2012) Host specificity determinants as a genetic continuum. Trends Microbiol 20: 88–93. 22196375 10.1016/j.tim.2011.11.006

[10] Nguyen DH, Ludgate L, Hu J (2008) Hepatitis B virus-cell interactions and pathogenesis. J Cell Physiol 216: 289–294. 18302164 10.1002/jcp.21416 PMC4386630

[11] Hu J, Seeger C (1996) Hsp90 is required for the activity of a hepatitis B virus reverse transcriptase. Proc Natl Acad Sci U S A 93: 1060–1064. 8577714 10.1073/pnas.93.3.1060 PMC40030

[12] Reese V, Ondracek C, Rushing C, Li L, Oropeza CE, et al (2011) Multiple nuclear receptors may regulate hepatitis B virus biosynthesis during development. Int J Biochem Cell Biol 43: 230–237. 19941970 10.1016/j.biocel.2009.11.016 PMC3773232

[13] Tang H, Banks KE, Anderson AL, McLachlan A (2001) Hepatitis B virus transcription and replication. Drug News Perspect 14: 325–334. 12813595

[14] Wang SH, Yeh SH, Lin WH, Yeh KH, Yuan Q, et al (2012) Estrogen receptor alpha represses transcription of HBV genes via interaction with hepatocyte nuclear factor 4alpha. Gastroenterology 142: 989–e984, 989-998, e984. 22240483 10.1053/j.gastro.2011.12.045

[15] Isogawa M, Robek MD, Furuichi Y, Chisari FV (2005) Toll-like receptor signaling inhibits hepatitis B virus replication in vivo. J Virol 79: 7269–7272. 15890966 10.1128/JVI.79.11.7269-7272.2005 PMC1112123

[16] Robek MD, Boyd BS, Chisari FV (2005) Lambda interferon inhibits hepatitis B and C virus replication. J Virol 79: 3851–3854. 15731279 10.1128/JVI.79.6.3851-3854.2005 PMC1075734

[17] Robek MD, Boyd BS, Wieland SF, Chisari FV (2004) Signal transduction pathways that inhibit hepatitis B virus replication. Proc Natl Acad Sci U S A 101: 1743–1747. 14757813 10.1073/pnas.0308340100 PMC341846

[18] Kimura K, Kakimi K, Wieland S, Guidotti LG, Chisari FV (2002) Interleukin-18 inhibits hepatitis B virus replication in the livers of transgenic mice. J Virol 76: 10702–10707. 12368312 10.1128/JVI.76.21.10702-10707.2002 PMC136645

[19] Puro R, Schneider RJ (2007) Tumor necrosis factor activates a conserved innate antiviral response to hepatitis B virus that destabilizes nucleocapsids and reduces nuclear viral DNA. J Virol 81: 7351–7362. 17475655 10.1128/JVI.00554-07 PMC1933346

[20] Hosel M, Quasdorff M, Wiegmann K, Webb D, Zedler U, et al (2009) Not interferon, but interleukin-6 controls early gene expression in hepatitis B virus infection. Hepatology 50: 1773–1782. 19937696 10.1002/hep.23226

[21] Chisari FV, Isogawa M, Wieland SF (2010) Pathogenesis of hepatitis B virus infection. Pathol Biol (Paris) 58: 258–266. 20116937 10.1016/j.patbio.2009.11.001 PMC2888709

[22] Guidotti LG, Chisari FV (2006) Immunobiology and pathogenesis of viral hepatitis. Annu Rev Pathol 1: 23–61. 18039107 10.1146/annurev.pathol.1.110304.100230

[23] Kawai T, Akira S (2006) Innate immune recognition of viral infection. Nat Immunol 7: 131–137. 16424890 10.1038/ni1303

[24] Sadler AJ, Williams BR (2008) Interferon-inducible antiviral effectors. Nat Rev Immunol 8: 559–568. 18575461 10.1038/nri2314 PMC2522268

[25] Wieland SF, Chisari FV (2005) Stealth and cunning: hepatitis B and hepatitis C viruses. J Virol 79: 9369–9380. 16014900 10.1128/JVI.79.15.9369-9380.2005 PMC1181548

[26] Wang H, Ryu WS (2010) Hepatitis B virus polymerase blocks pattern recognition receptor signaling via interaction with DDX3: implications for immune evasion. PLoS Pathog 6: e1000986. 20657822 10.1371/journal.ppat.1000986 PMC2904777

[27] Lang T, Lo C, Skinner N, Locarnini S, Visvanathan K, et al (2011) The hepatitis B e antigen (HBeAg) targets and suppresses activation of the toll-like receptor signaling pathway. J Hepatol 55: 762–769. 21334391 10.1016/j.jhep.2010.12.042

[28] Yu S, Chen J, Wu M, Chen H, Kato N, et al (2010) Hepatitis B virus polymerase inhibits RIG-I- and Toll-like receptor 3-mediated beta interferon induction in human hepatocytes through interference with interferon regulatory factor 3 activation and dampening of the interaction between TBK1/IKKepsilon and DDX3. J Gen Virol 91: 2080–2090. 20375222 10.1099/vir.0.020552-0

[29] Kumar M, Jung SY, Hodgson AJ, Madden CR, Qin J, et al (2011) Hepatitis B virus regulatory HBx protein binds to adaptor protein IPS-1 and inhibits the activation of beta interferon. J Virol 85: 987–995. 21068253 10.1128/JVI.01825-10 PMC3020017

[30] Scaglione SJ, Lok AS (2012) Effectiveness of hepatitis B treatment in clinical practice. Gastroenterology 142: 1360–e1361, 1360-1368, e1361. 22537444 10.1053/j.gastro.2012.01.044

[31] Mao R, Zhang J, Jiang D, Cai D, Levy JM, et al (2011) Indoleamine 2,3-dioxygenase mediates the antiviral effect of gamma interferon against hepatitis B virus in human hepatocyte-derived cells. J Virol 85: 1048–1057. 21084489 10.1128/JVI.01998-10 PMC3019998

[32] Uprichard SL, Wieland SF, Althage A, Chisari FV (2003) Transcriptional and posttranscriptional control of hepatitis B virus gene expression. Proc Natl Acad Sci U S A 100: 1310–1315. 12552098 10.1073/pnas.252773599 PMC298769

[33] Rang A, Gunther S, Will H (1999) Effect of interferon alpha on hepatitis B virus replication and gene expression in transiently transfected human hepatoma cells. J Hepatol 31: 791–799. 10580575 10.1016/s0168-8278(99)80279-7

[34] Wieland SF, Eustaquio A, Whitten-Bauer C, Boyd B, Chisari FV (2005) Interferon prevents formation of replication-competent hepatitis B virus RNA-containing nucleocapsids. Proc Natl Acad Sci U S A 102: 9913–9917. 15994231 10.1073/pnas.0504273102 PMC1175012

[35] Robek MD, Wieland SF, Chisari FV (2002) Inhibition of hepatitis B virus replication by interferon requires proteasome activity. J Virol 76: 3570–3574. 11884582 10.1128/JVI.76.7.3570-3574.2002 PMC136040

[36] Xu C, Guo H, Pan XB, Mao R, Yu W, et al (2010) Interferons accelerate decay of replication-competent nucleocapsids of hepatitis B virus. J Virol 84: 9332–9340. 20610715 10.1128/JVI.00918-10 PMC2937652

[37] Gao G, Guo X, Goff SP (2002) Inhibition of retroviral RNA production by ZAP, a CCCH-type zinc finger protein. Science 297: 1703–1706. 12215647 10.1126/science.1074276

[38] Bick MJ, Carroll JW, Gao G, Goff SP, Rice CM, et al (2003) Expression of the zinc-finger antiviral protein inhibits alphavirus replication. J Virol 77: 11555–11562. 14557641 10.1128/JVI.77.21.11555-11562.2003 PMC229374

[39] Muller S, Moller P, Bick MJ, Wurr S, Becker S, et al (2007) Inhibition of filovirus replication by the zinc finger antiviral protein. J Virol 81: 2391–2400. 17182693 10.1128/JVI.01601-06 PMC1865956

[40] Zhang Y, Burke CW, Ryman KD, Klimstra WB (2007) Identification and characterization of interferon-induced proteins that inhibit alphavirus replication. J Virol 81: 11246–11255. 17686841 10.1128/JVI.01282-07 PMC2045553

[41] Kerns JA, Emerman M, Malik HS (2008) Positive selection and increased antiviral activity associated with the PARP-containing isoform of human zinc-finger antiviral protein. PLoS Genet 4: e21. 18225958 10.1371/journal.pgen.0040021 PMC2213710

[42] Zhu Y, Chen G, Lv F, Wang X, Ji X, et al (2011) Zinc-finger antiviral protein inhibits HIV-1 infection by selectively targeting multiply spliced viral mRNAs for degradation. Proc Natl Acad Sci U S A 108: 15834–15839. 21876179 10.1073/pnas.1101676108 PMC3179061

[43] Wang X, Tu F, Zhu Y, Gao G (2012) Zinc-finger antiviral protein inhibits XMRV infection. PLoS One 7: e39159. 22720057 10.1371/journal.pone.0039159 PMC3376128

[44] Guo X, Carroll JW, Macdonald MR, Goff SP, Gao G (2004) The zinc finger antiviral protein directly binds to specific viral mRNAs through the CCCH zinc finger motifs. J Virol 78: 12781–12787. 15542630 10.1128/JVI.78.23.12781-12787.2004 PMC525010

[45] Guo X, Ma J, Sun J, Gao G (2007) The zinc-finger antiviral protein recruits the RNA processing exosome to degrade the target mRNA. Proc Natl Acad Sci U S A 104: 151–156. 17185417 10.1073/pnas.0607063104 PMC1765426

[46] Zhu Y, Gao G (2008) ZAP-mediated mRNA degradation. RNA Biol 5: 65–67. 18418085 10.4161/rna.5.2.6044

[47] Xuan Y, Liu L, Shen S, Deng H, Gao G (2012) Zinc finger antiviral protein inhibits murine gammaherpesvirus 68 m2 expression and regulates viral latency in cultured cells. J Virol 86: 12431–12434. 22951821 10.1128/JVI.01514-12 PMC3486457

[48] Rothnie HM, Chapdelaine Y, Hohn T (1994) Pararetroviruses and retroviruses: a comparative review of viral structure and gene expression strategies. Adv Virus Res 44: 1–67. 7817872 10.1016/s0065-3527(08)60327-9

[49] MacDonald MR, Machlin ES, Albin OR, Levy DE (2007) The zinc finger antiviral protein acts synergistically with an interferon-induced factor for maximal activity against alphaviruses. J Virol 81: 13509–13518. 17928353 10.1128/JVI.00402-07 PMC2168828

[50] Marcello T, Grakoui A, Barba-Spaeth G, Machlin ES, Kotenko SV, et al (2006) Interferons alpha and lambda inhibit hepatitis C virus replication with distinct signal transduction and gene regulation kinetics. Gastroenterology 131: 1887–1898. 17087946 10.1053/j.gastro.2006.09.052

[51] Wang N, Dong Q, Li J, Jangra RK, Fan M, et al (2010) Viral induction of the zinc finger antiviral protein is IRF3-dependent but NF-kappaB-independent. J Biol Chem 285: 6080–6090. 20048147 10.1074/jbc.M109.054486 PMC2825402

[52] Reiterer G, MacDonald R, Browning JD, Morrow J, Matveev SV, et al (2005) Zinc deficiency increases plasma lipids and atherosclerotic markers in LDL-receptor-deficient mice. J Nutr 135: 2114–2118. 16140885 10.1093/jn/135.9.2114

[53] Guo H, Jiang D, Ma D, Chang J, Dougherty AM, et al (2009) Activation of pattern recognition receptor-mediated innate immunity inhibits the replication of hepatitis B virus in human hepatocyte-derived cells. J Virol 83: 847–858. 18971270 10.1128/JVI.02008-08 PMC2612386

[54] Abraham TM, Lewellyn EB, Haines KM, Loeb DD (2008) Characterization of the contribution of spliced RNAs of hepatitis B virus to DNA synthesis in transfected cultures of Huh7 and HepG2 cells. Virology 379: 30–37. 18657840 10.1016/j.virol.2008.06.021 PMC2603046

[55] Liu L, Chen G, Ji X, Gao G (2004) ZAP is a CRM1-dependent nucleocytoplasmic shuttling protein. Biochem Biophys Res Commun 321: 517–523. 15358138 10.1016/j.bbrc.2004.06.174

[56] Jeong MS, Kim EJ, Jang SB (2010) Expression and RNA-binding of human zinc-finger antiviral protein. Biochem Biophys Res Commun 396: 696–702. 20451500 10.1016/j.bbrc.2010.04.164

[57] Chen S, Xu Y, Zhang K, Wang X, Sun J, et al (2012) Structure of N-terminal domain of ZAP indicates how a zinc-finger protein recognizes complex RNA. Nat Struct Mol Biol 19: 430–435. 22407013 10.1038/nsmb.2243

[58] Lai WS, Kennington EA, Blackshear PJ (2002) Interactions of CCCH zinc finger proteins with mRNA: non-binding tristetraprolin mutants exert an inhibitory effect on degradation of AU-rich element-containing mRNAs. J Biol Chem 277: 9606–9613. 11782475 10.1074/jbc.M110395200

[59] Lai WS, Blackshear PJ (2001) Interactions of CCCH zinc finger proteins with mRNA: tristetraprolin-mediated AU-rich element-dependent mRNA degradation can occur in the absence of a poly(A) tail. J Biol Chem 276: 23144–23154. 11279239 10.1074/jbc.M100680200

[60] Lai WS, Carballo E, Thorn JM, Kennington EA, Blackshear PJ (2000) Interactions of CCCH zinc finger proteins with mRNA. Binding of tristetraprolin-related zinc finger proteins to Au-rich elements and destabilization of mRNA. J Biol Chem 275: 17827–17837. 10751406 10.1074/jbc.M001696200

[61] Karki S, Li MM, Schoggins JW, Tian S, Rice CM, et al (2012) Multiple interferon stimulated genes synergize with the zinc finger antiviral protein to mediate anti-alphavirus activity. PLoS One 7: e37398. 22615998 10.1371/journal.pone.0037398 PMC3353916

[62] Hayakawa S, Shiratori S, Yamato H, Kameyama T, Kitatsuji C, et al (2011) ZAPS is a potent stimulator of signaling mediated by the RNA helicase RIG-I during antiviral responses. Nat Immunol 12: 37–44. 21102435 10.1038/ni.1963

[63] Chisari FV (1993) The role of cytotoxic T lymphocytes and inflammatory cytokines in the pathogenesis of acute viral hepatitis. Gastroenterol Jpn 28 Suppl 4: 2–6 discussion 17-19. 8486226 10.1007/BF02782879

[64] Wieland S, Thimme R, Purcell RH, Chisari FV (2004) Genomic analysis of the host response to hepatitis B virus infection. Proc Natl Acad Sci U S A 101: 6669–6674. 15100412 10.1073/pnas.0401771101 PMC404103

[65] Wieland SF, Vega RG, Muller R, Evans CF, Hilbush B, et al (2003) Searching for interferon-induced genes that inhibit hepatitis B virus replication in transgenic mouse hepatocytes. J Virol 77: 1227–1236. 12502840 10.1128/JVI.77.2.1227-1236.2003 PMC140855

[66] Lok AS, Heathcote EJ, Hoofnagle JH (2001) Management of hepatitis B: 2000–summary of a workshop. Gastroenterology 120: 1828–1853. 11375963 10.1053/gast.2001.24839

[67] Zoulim F, Mason WS (2012) Reasons to consider earlier treatment of chronic HBV infections. Gut 61: 333–336. 22147510 10.1136/gutjnl-2011-300937

[68] Kawai T, Takahashi K, Sato S, Coban C, Kumar H, et al (2005) IPS-1, an adaptor triggering RIG-I- and Mda5-mediated type I interferon induction. Nat Immunol 6: 981–988. 16127453 10.1038/ni1243

[69] Seth RB, Sun L, Ea CK, Chen ZJ (2005) Identification and characterization of MAVS, a mitochondrial antiviral signaling protein that activates NF-kappaB and IRF 3. Cell 122: 669–682. 16125763 10.1016/j.cell.2005.08.012

[70] Meylan E, Curran J, Hofmann K, Moradpour D, Binder M, et al (2005) Cardif is an adaptor protein in the RIG-I antiviral pathway and is targeted by hepatitis C virus. Nature 437: 1167–1172. 16177806 10.1038/nature04193

[71] Xu LG, Wang YY, Han KJ, Li LY, Zhai Z, et al (2005) VISA is an adapter protein required for virus-triggered IFN-beta signaling. Mol Cell 19: 727–740. 16153868 10.1016/j.molcel.2005.08.014

[72] Kawai T, Akira S (2007) Antiviral signaling through pattern recognition receptors. J Biochem 141: 137–145. 17190786 10.1093/jb/mvm032

[73] Takeuchi O, Akira S (2008) MDA5/RIG-I and virus recognition. Curr Opin Immunol 20: 17–22. 18272355 10.1016/j.coi.2008.01.002

[74] Law LM, Albin OR, Carroll JW, Jones CT, Rice CM, et al (2010) Identification of a dominant negative inhibitor of human zinc finger antiviral protein reveals a functional endogenous pool and critical homotypic interactions. J Virol 84: 4504–4512. 20181706 10.1128/JVI.02018-09 PMC2863759

[75] Huang Z, Wang X, Gao G (2010) Analyses of SELEX-derived ZAP-binding RNA aptamers suggest that the binding specificity is determined by both structure and sequence of the RNA. Protein Cell 1: 752–759. 21203916 10.1007/s13238-010-0096-9 PMC4875198

[76] Hu J, Lin L (2009) RNA-protein interactions in hepadnavirus reverse transcription. Front Biosci 14: 1606–1618. 10.2741/3328 PMC3611959 19273150

[77] Kim VN (2005) MicroRNA biogenesis: coordinated cropping and dicing. Nat Rev Mol Cell Biol 6: 376–385. 15852042 10.1038/nrm1644

[78] Ye P, Liu S, Zhu Y, Chen G, Gao G (2010) DEXH-Box protein DHX30 is required for optimal function of the zinc-finger antiviral protein. Protein Cell 1: 956–964. 21204022 10.1007/s13238-010-0117-8 PMC4875121

[79] Chen G, Guo X, Lv F, Xu Y, Gao G (2008) p72 DEAD box RNA helicase is required for optimal function of the zinc-finger antiviral protein. Proc Natl Acad Sci U S A 105: 4352–4357. 18334637 10.1073/pnas.0712276105 PMC2393818

[80] Guo H, Jiang D, Zhou T, Cuconati A, Block TM, et al (2007) Characterization of the intracellular deproteinized relaxed circular DNA of hepatitis B virus: an intermediate of covalently closed circular DNA formation. J Virol 81: 12472–12484. 17804499 10.1128/JVI.01123-07 PMC2169032

[81] Guo H, Zhou T, Jiang D, Cuconati A, Xiao GH, et al (2007) Regulation of hepatitis B virus replication by the phosphatidylinositol 3-kinase-akt signal transduction pathway. J Virol 81: 10072–10080. 17609269 10.1128/JVI.00541-07 PMC2045390

[82] Schormann W, Kraft A, Ponsel D, Bruss V (2006) Hepatitis B virus particle formation in the absence of pregenomic RNA and reverse transcriptase. J Virol 80: 4187–4190. 16571836 10.1128/JVI.80.8.4187-4190.2006 PMC1440432

[83] Galibert F, Mandart E, Fitoussi F, Charnay P (1979) Nucleotide sequence of the hepatitis B virus genome (subtype ayw) cloned in E.coli. Nature 281: 646–650. 399327 10.1038/281646a0

