# Supplementary figures and images for: Inhibition of Hepatitis B Virus Replication by the Host Zinc Finger Antiviral Protein
Source: PLoS Pathog. 2013 Jul 11;9(7):e1003494. doi: 10.1371/journal.ppat.1003494 (PMC3708887; doi:10.1371/journal.ppat.1003494)

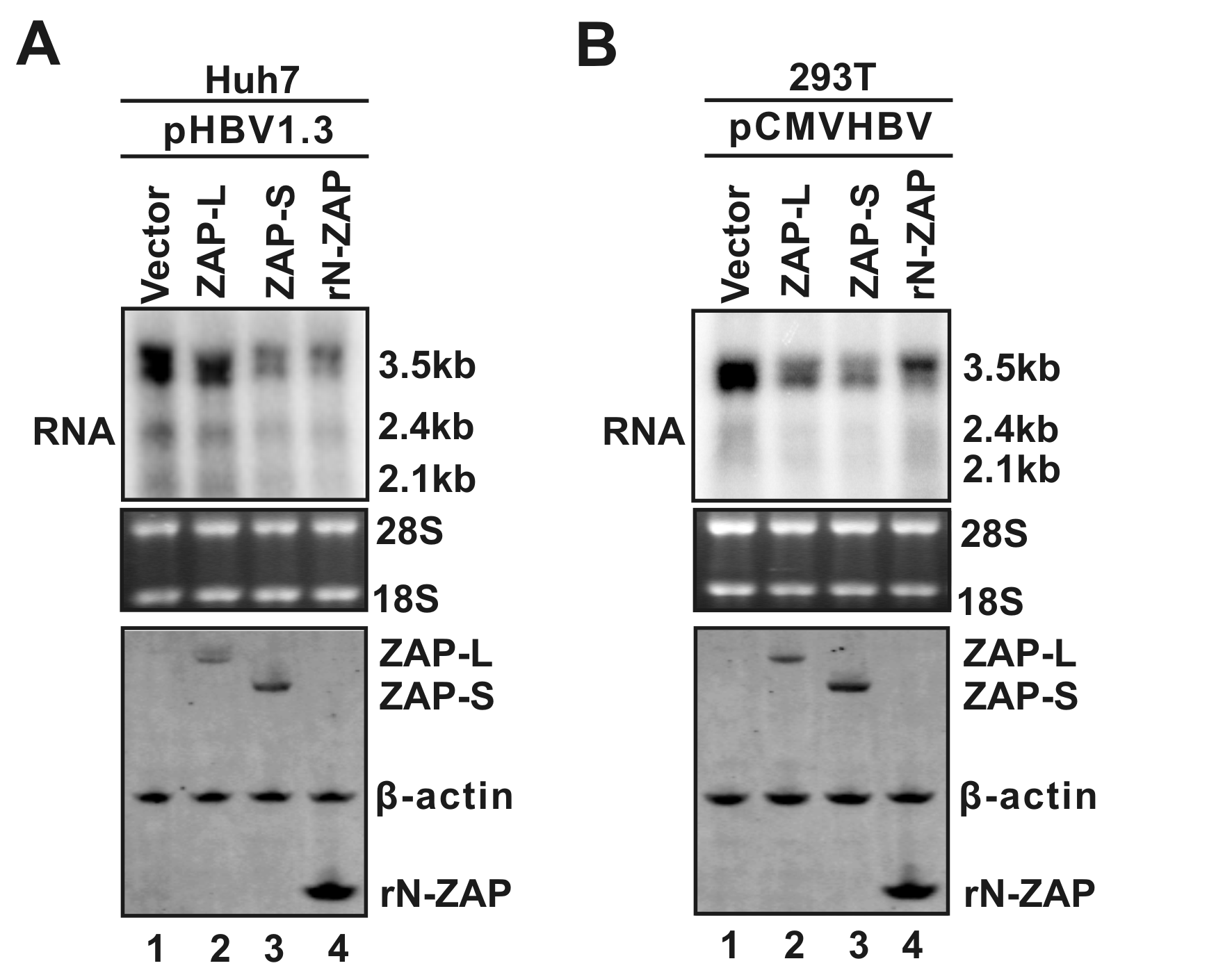

Supplement: Figure S1 — Expression of ZAP reduces the steady state levels of HBV RNA in Huh7 and 293T cells. (A) Human hepatoma Huh7 cells in 35 mm dishes were cotransfected with 2 µg of plasmid pHBV1.3 plus equal amounts of either control vector, or plasmid expressing HA-tagged hZAP-L, hZAP-S, and rN-ZAP, respectively. Cells were harvested at day 4 post transfection and viral RNAs were analyzed by Northern blot assay (top panel). Expression of ZAP was revealed by Western blot analysis with HA antibodies (bottom panel). The levels of β-actin were probed simultaneously on the same blot as loading controls. (B) Human embryonic kidney 293T cells were transfected and analyzed in the same way as Huh7 cells, except for the plasmid pHBV1.3 was replaced by pCMVHBV to bypass the requirement of liver specific transcription factors for HBV pgRNA transcription. (TIF) [file ppat.1003494.s001.tif]

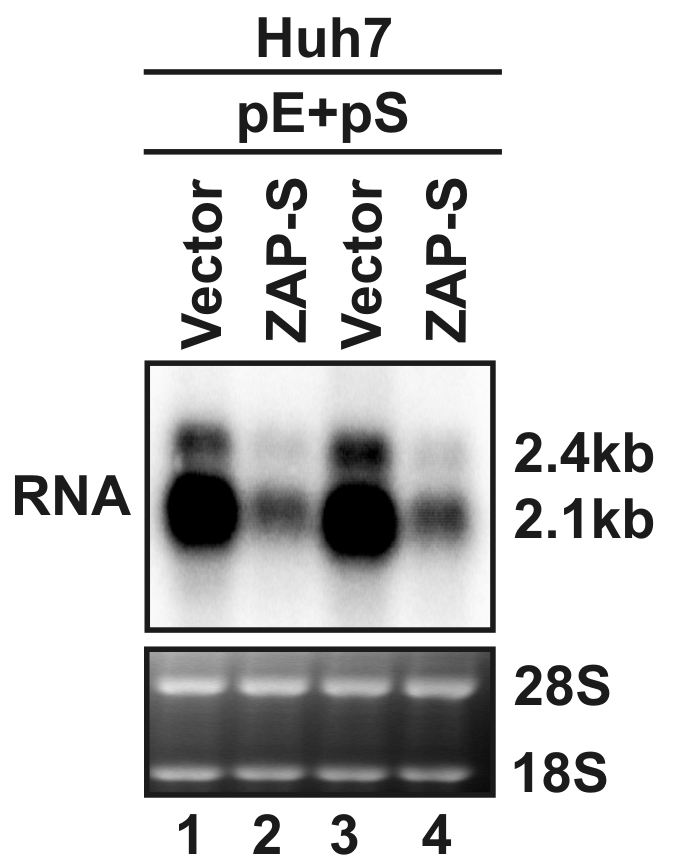

Supplement: Figure S2 — ZAP expression reduces the levels of HBV subgenomic RNA. HepG2 cells in 35 mm dishes were cotransfected with plasmids pE and pS (1 µg of each), plus 2 µg of control vector, or plasmid ZAP-S. Four days later, HBV subgenomic RNA (2.4 kb and 2.1 kb in length) were detected by Northern blot hybridization. Results from duplicate experiments are presented. (TIF) [file ppat.1003494.s002.tif]

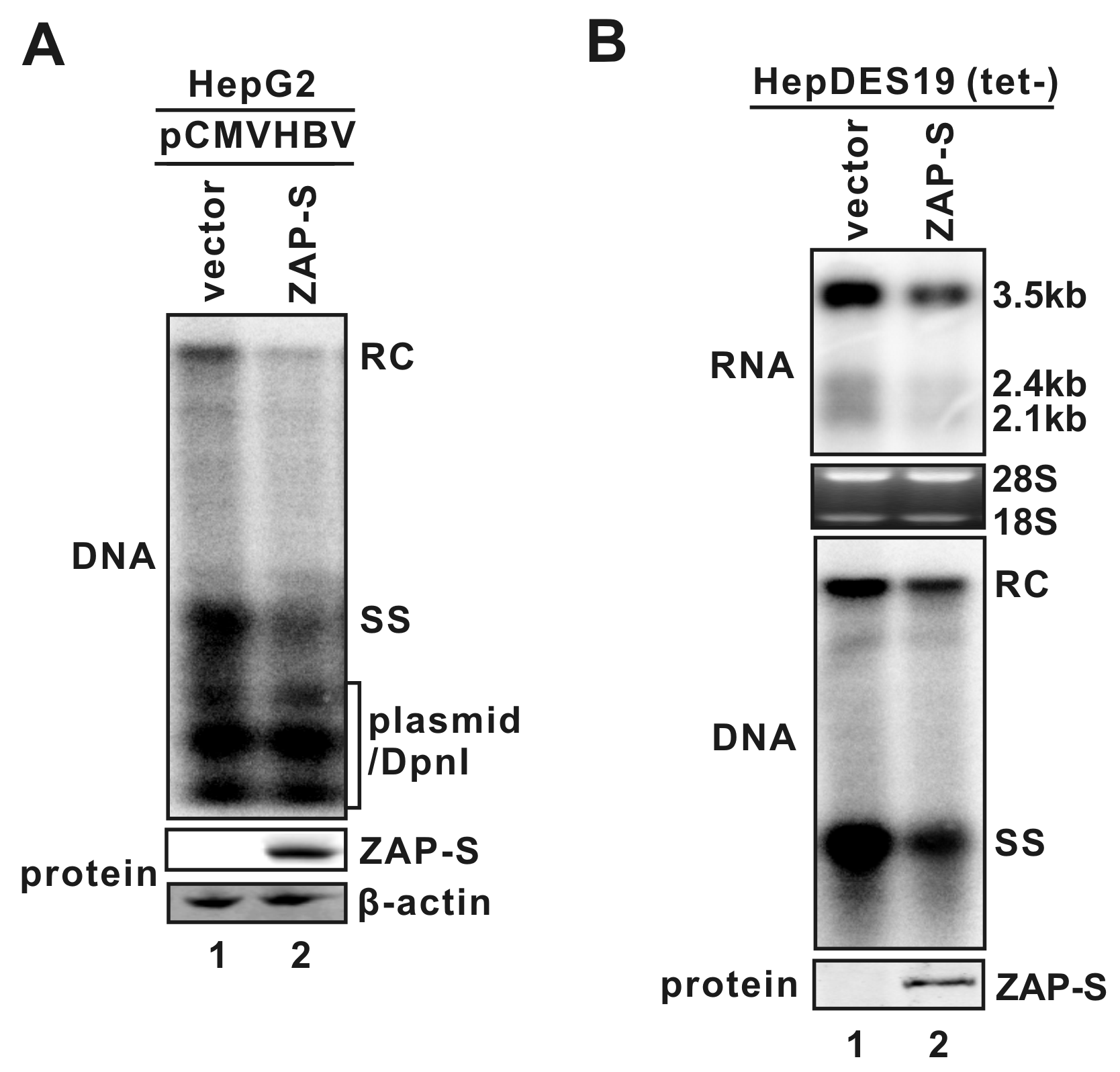

Supplement: Figure S3 — ZAP does not reduce the levels of HBV RNA transcription template. (A) ZAP does not promote the elimination of transfected HBV plasmid DNA. HepG2 cells in 35 mm dishes were cotransfected with 2 µg of plasmid pCMVHBV and 2 µg of control vector or plasmid that expresses ZAP-S. The cells were harvested at day 5 post transfection and viral DNA was analyzed by hybridization. During cytoplasmic HBV DNA extraction, DNase I digestion of input HBV plasmid DNA in cell lysates was omitted prior to SDS/pronase treatment, and the DNA samples were incubated with DpnI restriction enzyme to digest the bacteria-derived plasmid DNA at methylated DpnI cleavage sites. The DpnI-restricted HBV plasmid DNA fragments that migrated underneath the viral single strand DNA were revealed by electrophoresis and Southern hybridization. Expression of ZAP-S was detected by Western blot with antibodies against HA-tag. β-actin served as loading control. (B) Expression of ZAP reduces HBV RNA and DNA in stably transfected HBV cell line. Tetracycline inducible (tet-off) HBV stable cell line, HepDES19 cells, were cultured in 35 mm dish, followed by tet withdrawal and transfection of 4 µg of control vector or plasmid ZAP-S. Five days later, viral nucleic acids and ZAP-S expression were analyzed. (TIF) [file ppat.1003494.s003.tif]

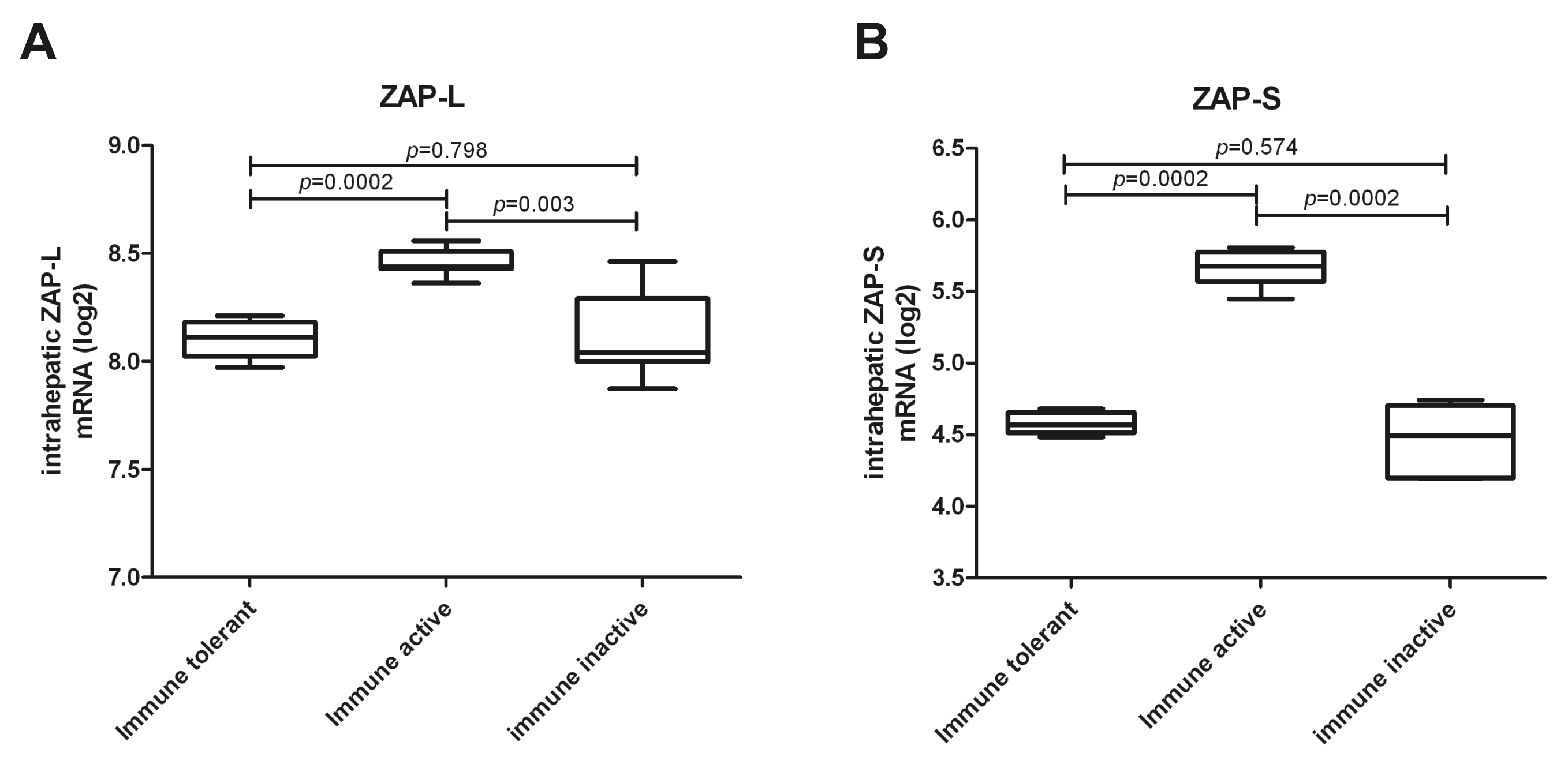

Supplement: Figure S4 — Intrahepatic mRNA levels of ZAP in chronic hepatitis B patients. Twenty-four patients were divided into three groups, specifically immune tolerant phase (8 patients), immune active phase (8 patients), and inactive carrier phase (8 patients) (Table S1). The mRNA levels of ZAP-L (A) and ZAP-S (B) from individual liver biopsy samples in each group (X-axis) were measured by gene chip analysis and plotted as log2 value with mean ± SD (Y-axis). p<0.05 is considered as significant. (TIF) [file ppat.1003494.s004.tif]

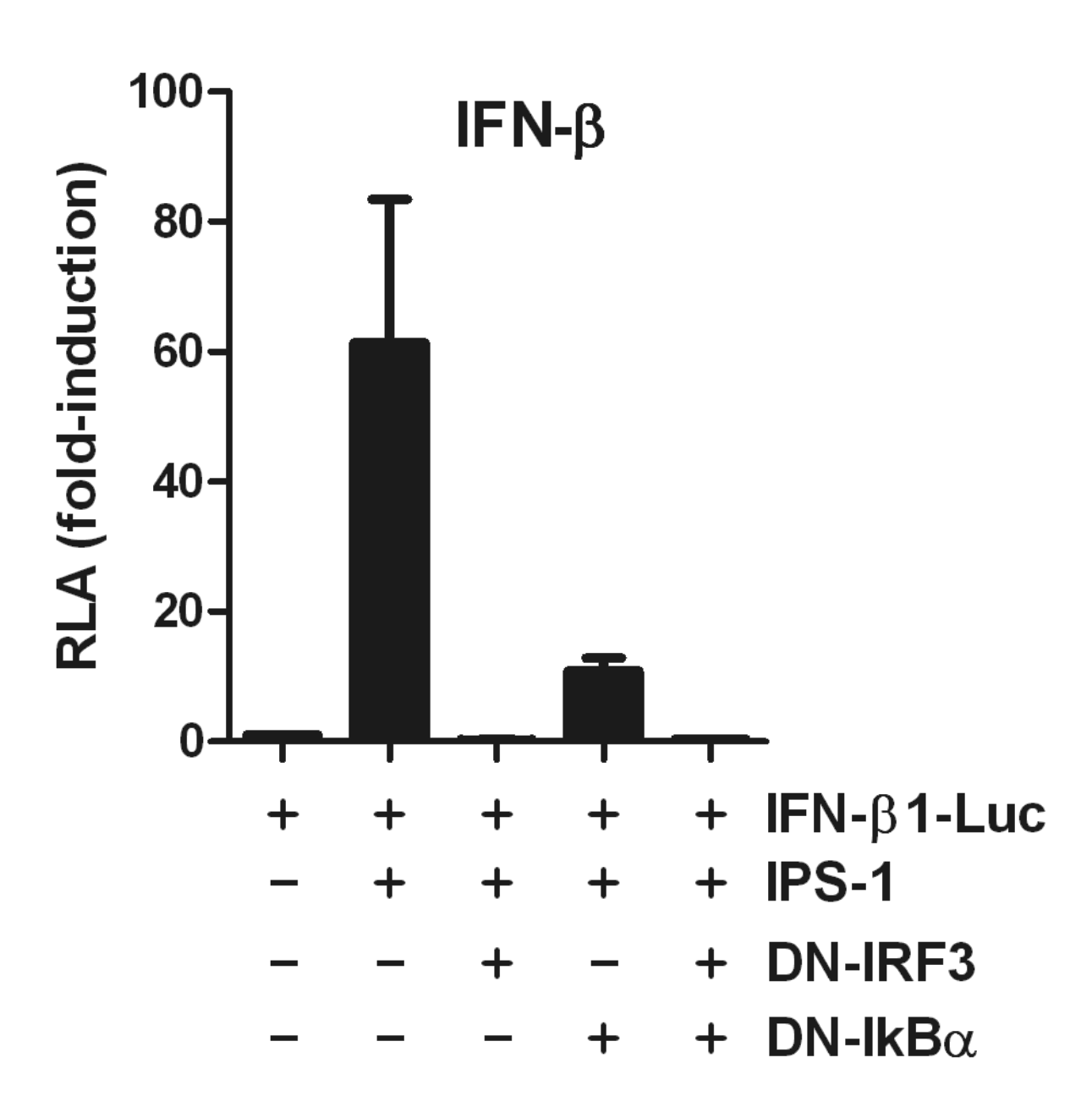

Supplement: Figure S5 — Expression of IPS-1 stimulates IFN-β promoter activity through IRF3 and NF-κB pathways. HepG2 cells were transfected with IFN-β1 promoter luciferase reporter plasmid IFN-β1-Luc and indicated plasmid expressing IPS-1, DN-IRF3, and DN-IκBα. Luciferase assay was performed at 48 h post transfection. Relative luciferase activity was expressed as fold induction (mean ± SD, n = 3) versus control. (TIF) [file ppat.1003494.s005.tif]

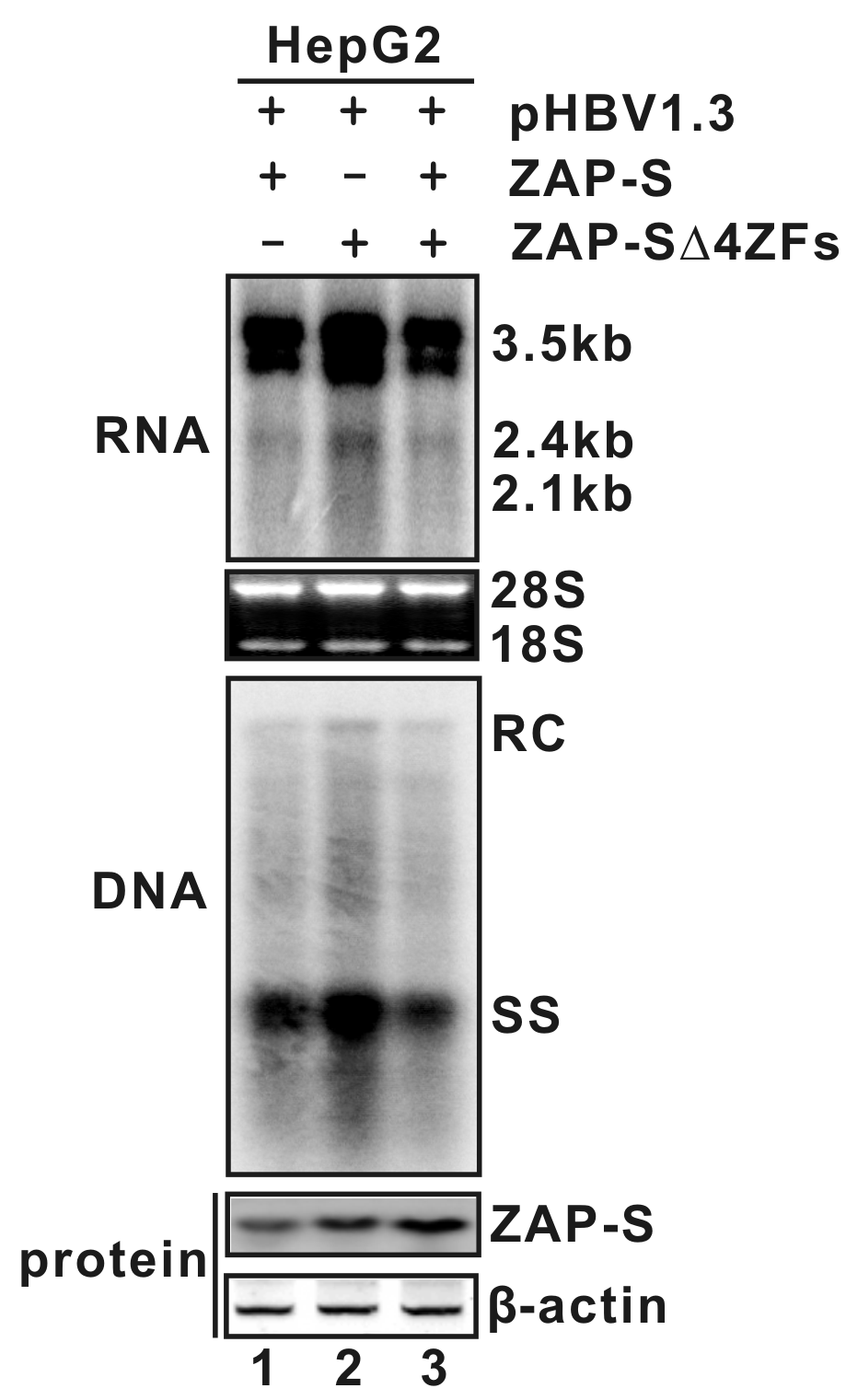

Supplement: Figure S6 — ZAP-SΔ4ZFs does not possess dominant negative effect on ZAP's antiviral activity. HepG2 cells were cotransfected with indicated plasmids for 5 days, followed by analyses of viral RNA, DNA, and ZAP-S expression. (TIF) [file ppat.1003494.s006.tif]

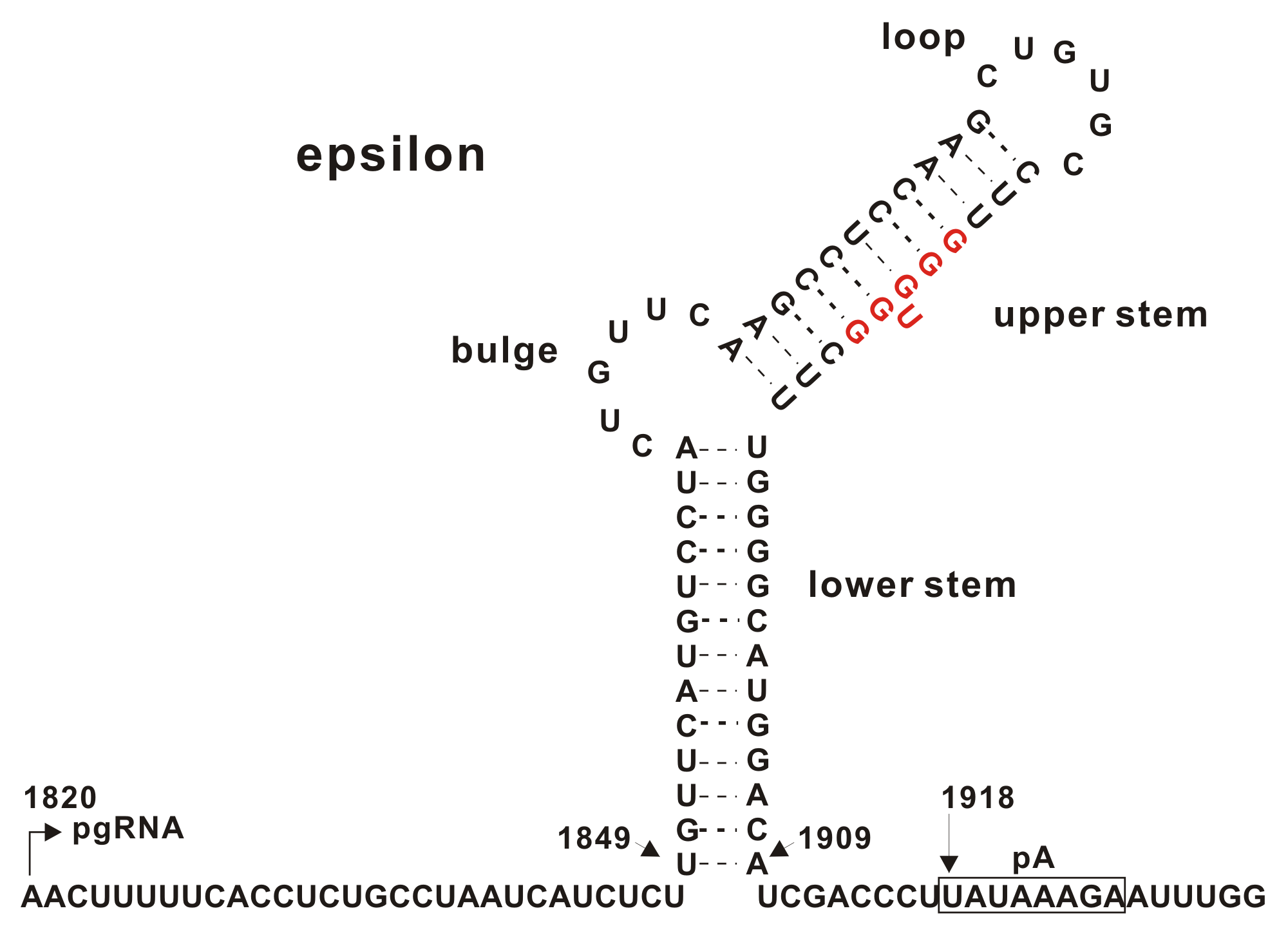

Supplement: Figure S7 — RNA sequences and predicted secondary structure of HBV ZRE. HBV terminal redundancy ranges from the initiation site (nt 1820) of pgRNA to the polyadenylation site (pA, nt 1918). Stem-loop structure (epsilon, ε) is illustrated according to the literature [7]. “GGGUGG” motif is highlighted in red. (TIF) [file ppat.1003494.s007.tif]

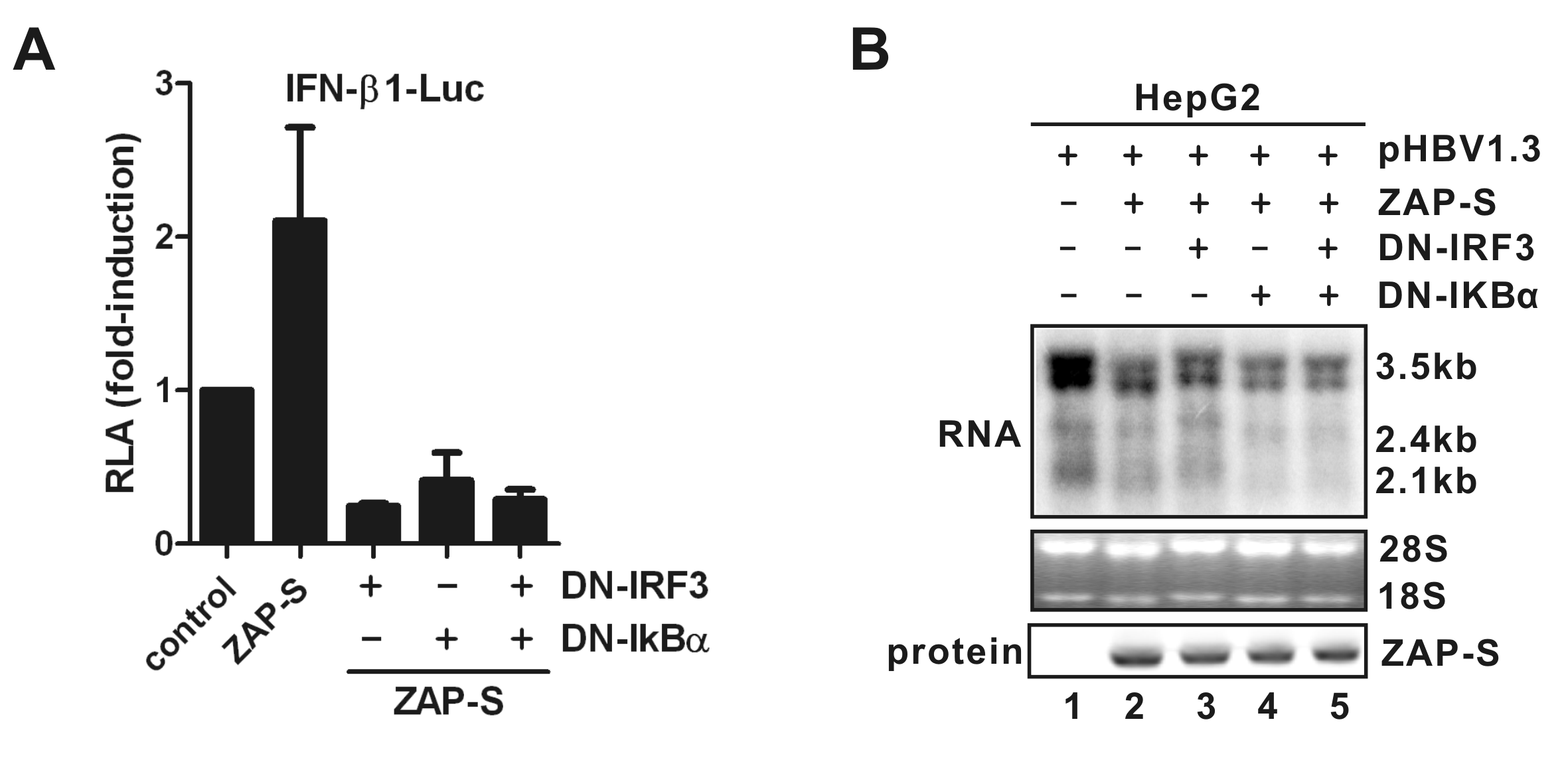

Supplement: Figure S8 — ZAP-mediated HBV RNA reduction is independent of IFN. (A) Overexpression of ZAP only slightly stimulates IFN-β promoter activity. HepG2 cells were transfected with indicated plasmids for 48 h. IFN-β1 promoter-driven luciferase activity was expressed as fold induction (mean ± SD, n = 3) over control. (B) Inhibition of IFN production does not rescue ZAP-mediated HBV RNA reduction. HepG2 cells in 35 mm dishes were transfected with 1 µg of each indicated plasmids, and control vector was supplemented to ensure that each transfection received the same amount (4 µg) of total transfected DNA. Cells were harvested at day 3 post transfection, HBV RNA and HA-tagged ZAP-S were analyzed by Northern hybridization and Western blot, respectively. (TIF) [file ppat.1003494.s008.tif]

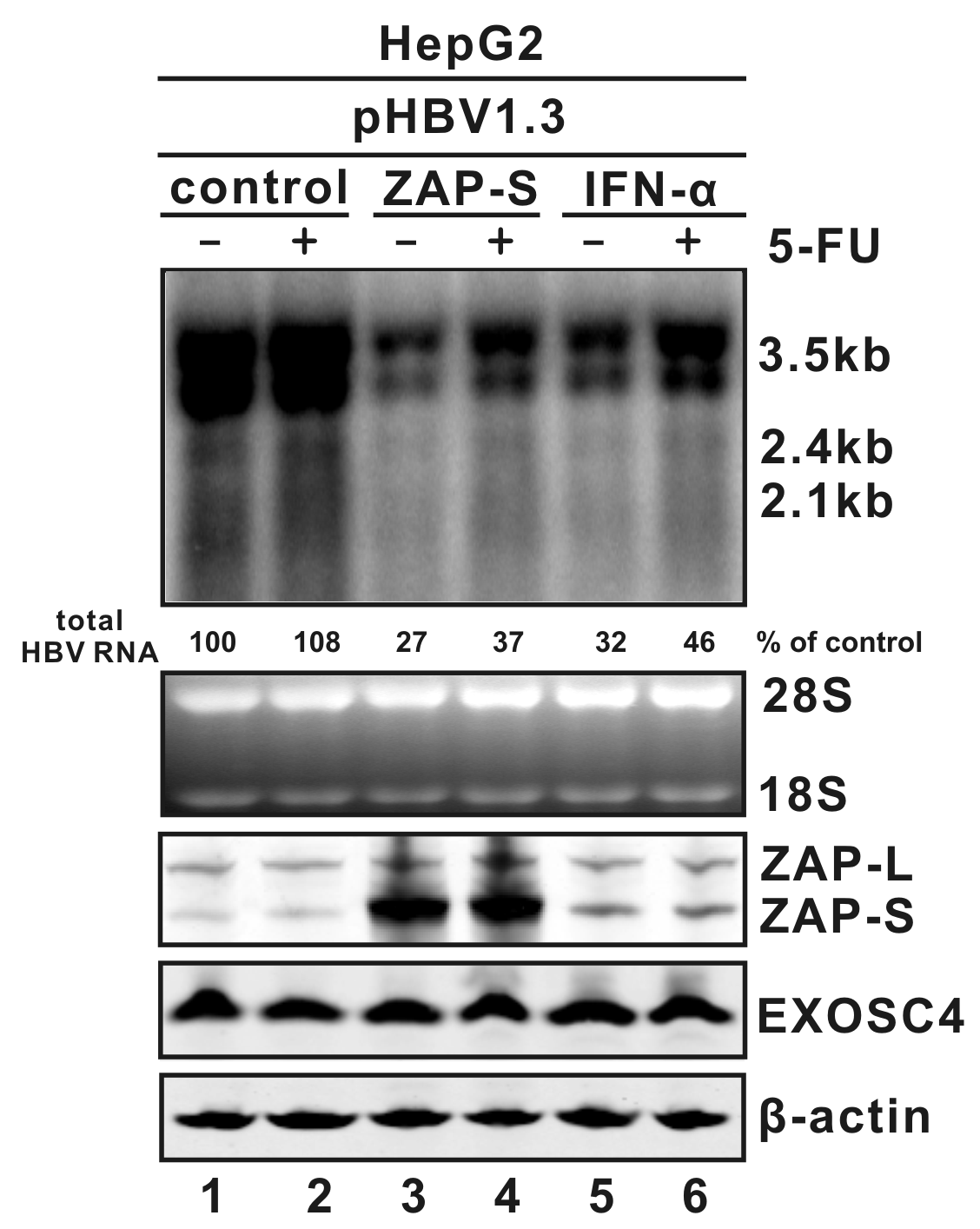

Supplement: Figure S9 — Exosome activity might be involved in ZAP-mediated HBV RNA reduction. HepG2 cells in 35 mm dishes were cotransfected with 2 µg of pHBV1.3 (lanes 1–6), and 2 µg of control vector (lanes 1, 2, 5, 6) or 2 µg of plasmid expressing HA-tagged ZAP-S (lanes 3 and 4). Twelve hours after transfection, cells were treated with 0.1% solvent DMSO (lanes 1, 3, 5), or 10 ng/ml 5-FU (lanes 2, 4, 6), two sets of pHBV1.3 and vector cotransfected cells were further treated with IFN-α (1,000 IU/ml) (lanes 5 and 6). The treatments were repeated every day for 3 days. Northern hybridization was performed to analyze HBV RNA (upper panel), and relative level of HBV RNA in each sample is expressed as the percentage of RNA level in the control samples (lanes 1), and is presented underneath the blot. The levels of ZAP and exosome subunit EXOSC4 were detected by Western blot, with β-actin serving as loading control. (TIF) [file ppat.1003494.s009.tif]
