# Supplementary material for: Inhibition of Hepatitis B Virus Replication by the Host Zinc Finger Antiviral Protein
Source: PLoS Pathog. 2013 Jul 11;9(7):e1003494. doi: 10.1371/journal.ppat.1003494 (PMC3708887; doi:10.1371/journal.ppat.1003494)
Supplement: Table S1 — The clinical characteristics of studied subjects. A total of 24 treatment-naïve chronic hepatitis B patients were classified into 3 phases of infection according to their serum ALT and HBV DNA levels, including immune tolerant phase (8 patients; normal ALT, HBV DNA>105 copies/ml), immune active phase (8 patients; elevated ALT, HBV DNA>105 copies/ml), and inactive phase (8 patients; normal ALT, HBV DNA<104 copies/ml). In each group, values are expressed as average (low-high) or number. Alanine transaminase (ALT); Hepatitis B surface antigen (HBsAg); Hepatitis B e antigen (HBeAg); Hepatitis B core antibody (Anti-HBc). (DOC) [file ppat.1003494.s010.doc]

**Table S1. The clinical characteristics of studied subjects**

| **Group** | **Immune tolerant** | **Immune active** | **Immune inactive** |
| --- | --- | --- | --- |
| **No. of patients** | 8 | 8 | 8 |
| **Age (yr )** | 23.0(18.1-30.2) | 30.0(24.0-36.2) | 38.2(35.0-44.6) |
| **Sex (male/female)** | 6/2 | 5/3 | 5/3 |
| **ALT (ULN)** | 0.6(0.4-0.9) | 2.6(2.1-7.8) | 0.5(0.3-0.8) |
| **Antiviral treatment** | 0 | 0 | 0 |
| **HBV DNA titer**  **(log_10_ copies/ml)** | 8.2(7.8-8.8) | 5.8(5.2-7.0) | 3.2(3.0-3.5) |
| **HBsAg positive** | 8 | 8 | 8 |
| **Anti-HBs positive** | 0 | 0 | 0 |
| **HBeAg positive** | 8 | 3 | 0 |
| **Anti-HBe positive** | 0 | 5 | 8 |
| **Anti-HBc positive** | 8 | 8 | 8 |
